# Supplementary material for: Robust links in photoactive covalent organic frameworks enable effective photocatalytic reactions under harsh conditions
Source: Nat Commun. 2024 Feb 10;15:1267. doi: 10.1038/s41467-024-45457-y (PMC10858907; doi:10.1038/s41467-024-45457-y)
Supplement: Supplementary file 1 — Supplementary Information [file 41467_2024_45457_MOESM1_ESM.pdf]

Supplementary Information

Robust links in photoactive covalent organic frameworks enable effective photocatalytic reactions under harsh conditions

Wang et al.

Supplementary Section 1: General Information

All starting materials are commercially available and were used as received unless specifically mentioned.

Powder X-ray diffraction (PXRD) data were collected at 40 kV and 200 mA on a SmartLab 9 kW X-ray diffractometer using Cu-*Kα* radiation (λ = 1.5418 Å) over 2θ range of 2.0-30.0° at room temperature. Fourier transform infrared (FT-IR) measurements were performed on an Avatar 370 FT-IR spectrometer. Solid-state ^{13}C (150.03 MHz) CP MAS NMR spectra were performed on a Bruker WB 600 spectrometer at 300 K with a 3.2 mm double channel probehead. Samples were carefully packed into a 3.2 mm cylindrical zirconia rotor with Kel-F caps. ^{13}C NMR spectra were originally calibrated with adamantane as the standard sample. ^{1}H NMR spectra were measured on a Bruker AVANCE-400 spectrometer (400 MHz ^{1}H) at ambient temperature. X-ray photoelectron spectroscopy (XPS) was measured on an Thermo Scientific K-Alpha. Scanning electron microscopy (SEM) images were recorded using a TESCAN MIRA LMS scanning electron microscope. Transmission electron microscopy (TEM) images were recorded using a FEI Tecnai F20. Thermogravimetric analyses (TGA) were carried out on a TGA 55 with a heating rate of 10 °C min^{-1} under N_2 flow (60 mL/min) from room temperature to 800 °C. Gas sorption analyses were conducted using Kubo-X1000 with extra-high pure gases. The fluorescence measurements were performed on the high efficiency integrated fluorescence spectrometer with the model of Horiba FluoroMax-4. UV-Vis spectra and UV-Vis DRS spectra were carried out on the UV-VIS-NIR spectrophotometer with the model of Hitachi U-4100. Electron paramagnetic resonance (EPR) spectra were conducted on a Bruker EMXplus-6/1 with Xe lamp (CEL-HXF300) as light source. DFT calculations of frontier molecular orbitals for the core molecular structures of TFPA-TAPT-COF-Q, TFPA-TAPT-COF, TFPA-TPB-COF-Q and TFPA-TPB-COF were obtained by ChemDraw 3D. Moreover, the calculations of the energy levels and optimization of COF fragments were based on density functional theory (DFT) by Gaussian 09W package. The molecular orbitals were obtained at the B3LYP/6-311+G(d,p) level. The electron excitation analysis was carried out by Multiwfn. The crystalline structures of all four COFs were built and refined using the software Materials Studio.

Photocatalysis Experiments

The experiments were carried out according to the reported method with slight modifications.[1] The substrate (0.2 mmol) was firstly dissolved in CH_3CN (2 mL) in a 10 mL Pyrex tube. The COFs (8 mg) were then added into the solution. The reaction mixture was magnetically stirred at 1500 rpm and illuminated with a LED light (total 24 W) in an open system with the additional electronic fan to warrant the reaction constantly operated at room temperature. TLC was used for monitoring the disappearance of reactants. After the reaction completed, the COF powder was separated from the reaction mixture by centrifugation and washed with CH_3CN several times. The products were collected by a 25 mL flask and concentrated by rotating distillation and then the internal standard (0.2 mmol) was added into the flask. ^1H NMR was utilized to identify the product, and calculate the yield and selectivity (See Supplementary Section 2 for each product). The COF powder for recycling reaction was collected by centrifugation at 5000 rpm and washed by fresh CH_3CN two times.

Photocatalysis Experiments for H_2O_2 Production

A photocatalysis reactor was charged with COF powder (10 mg) in water (20 mL) without or with a sacrificial reagent (BA, EtOH, IPA, or TEOA) (2 mL). The photocatalysis reactor was purged with pure O_2 for 10 min in each case. The photocatalytic H_2O_2 evolution experiments were performed on a 300 W Xe lamp with full-wave band light. After proper time, the solution (5 mL) was taken by a syringe. The remaining suspension was transferred to a 15 mL centrifuge tube, centrifuged at 8000rpm for 5 min, washed with ethanol three times, and then dried under vacuum at 60 °C for 1 h. The amount of H_2O_2 produced was analyzed with cerimetric titration. The obtained Ce^{4+} standard solution was mixed with the sample solution and detected by UV-Vis spectrophotometer.

Supplementary Tables:

Supplementary Table 1: Different quinoline-linkage COFs and their applications.

Entry	Quinoline-linked COFs	Applications	Ref.
1	TFPA-TAPT-COF-Q, TFPA-TPB-COF-Q	Photocatalysis under harsh condition (acid, base and strong oxidant)	This work
2	MF-1a, MF-1b, MF-1c, MF-1d, MF-1e	Methodology	2
3	P-StTaDm-COF	Methodology	3
4	COF-Ph, COF-$(CF_3)_2$	Adsorption and detection of pesticides	4
5	COF-Pyr, Co@COF-Pyr	Electrocatalyst for oxygen evolution reaction	5
6	COF-PI-2	Catalytic CO_2 cycloaddition via visible-light-induced photothermal conversion	6
7	QL-COF-1, QL-COF-2	Nanofiltration	7
8	TAPB-DMTP-PA-COF	Catalyze cascade reaction	8
9	$CQCOF_{TAPB\text{-}BPTA}$, $CQCOF_{TAPT\text{-}BPTA}$, $CQCOF_{Py\text{-}BPTA}$, $CQCOF_{NiP\text{-}BPTA}$	Dual-mode sensing of strongly acidic environments	9
10	$NQ\text{-}COF_{TfppyPh}$, $NQ\text{-}COF_{TfppyBp}$	Methodology and photocatalytic organic conversion	10
11	PYTRI-COF-2	Lithium storage	11
12	DMCR-1	Solar-driven H_2O_2 production	12
13	COF-BA	Sensitive and selective visualization of CH_3Hg^+	13
14	PMCR-1	Solar light driven H_2O_2 production and selective photocatalytic oxidations	14
15	$NQCOF_{A1}$	Methodology and photocatalytic organic conversion	15

Supplementary Table 2: Photocatalytic activity of COFs in methylphenyl sulfide oxidation.

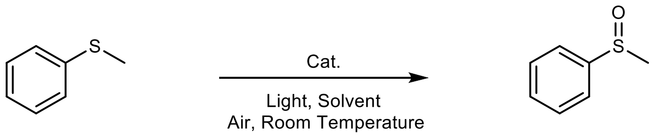

Entry	Light	Air	Catalyst	Time (h)	Solvent	Additive	Conv. (%)	Sel. (%)
1	-	+	TFPA-TAPT-COF-Q	2.5	MeCN	-	0	0
2	+	-	TFPA-TAPT-COF-Q	2.5	MeCN	-	0	0
3	+	+	-	2.5	MeCN	-	0	0
4	+	+	TFPA-TAPT-COF-Q	1.5	MeCN	-	53	100
5	+	+	TFPA-TAPT-COF-Q	2.5	MeCN	-	99	97
6	+	+	TFPA-TAPT-COF-Q	3.5	MeCN	-	100	85
7	+	+	TFPA-TAPT-COF-Q	4.5	MeCN	-	100	68
8	+	+	TFPA-TAPT-COF-Q	2.5	MeOH	-	84	26
9	+	+	TFPA-TAPT-COF-Q	2.5	$CHCl_3$	-	32	100
10	+	+	TFPA-TAPT-COF-Q	2.5	THF	-	6	100
11	+	+	TFPA-TAPT-COF	2.5	MeCN	-	99	93
12	+	+	TFPA-TPB-COF-Q	2.5	MeCN	-	99	98
13	+	+	TFPA-TPB-COF	2.5	MeCN	-	99	95
14	+	+	TFPA-TAPT-COF-Q	2.5	MeCN	BQ	3	100
15	+	+	TFPA-TAPT-COF-Q	2.5	MeCN	DABCO	85	100
16	+	+	TFPA-TAPT-COF-Q	2.5	MeCN	KI	11	5

Supplementary Table 3: Photocatalytic activity of TFPA-TAPT-COF-Q and TFPA-TAPT-COF in photocatalytic oxidation of sulfides.

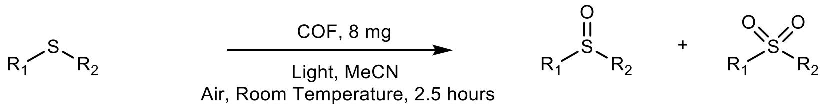

Entry	Substrate	TFPA-TAPT-COF-Q Conv. (%)	TFPA-TAPT-COF-Q Sel. (%)	TFPA-TAPT-COF Conv. (%)	TFPA-TAPT-COF Sel. (%)
1	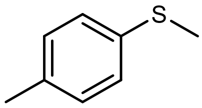	100	95	100	93
2	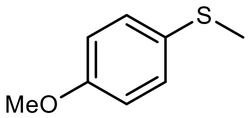	100	95	100	93
3	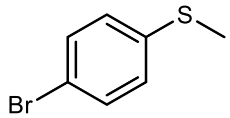	60	95	64	100
4	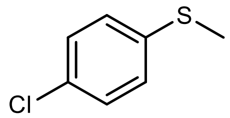	100	96	100	91
5	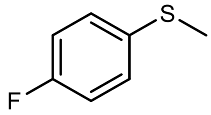	100	98	100	94
6	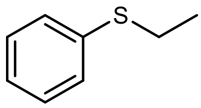	63	100	20	61
7	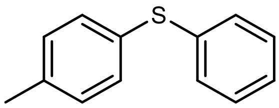	56	100	10	100
8	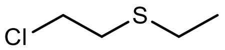	84	100	92	100

Supplementary Table 4: Photocatalytic activity of TFPA-TPB-COF-Q and TFPA-TPB-COF in photocatalytic oxidation of sulfides.

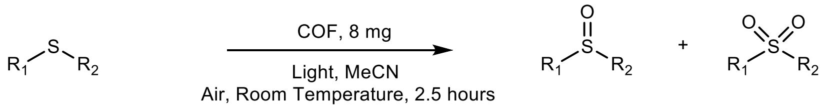

Entry	Substrate	TFPA-TPB-COF-Q Conv. (%)	TFPA-TPB-COF-Q Sel. (%)	TFPA-TPB-COF Conv. (%)	TFPA-TPB-COF Sel. (%)
1	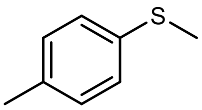	100	98	100	95
2	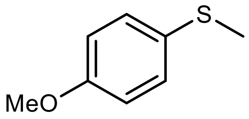	84	98	100	87
3	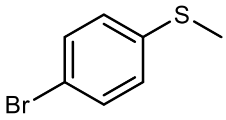	27	95	54	100
4	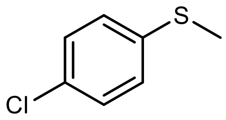	100	75	100	98
5	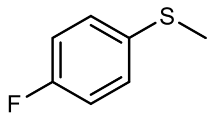	100	92	100	94
6	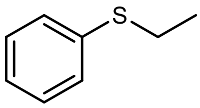	41	86	24	61
7	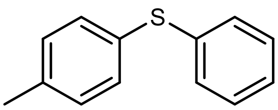	16	70	12	100
8	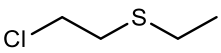	71	77	84	100

Supplementary Table 5: Photocatalytic activity of TFPA-TAPT-COF-Q in oxidative decarboxylation of 4-methoxyphenylacetic acid (TMG means 1,1,3,3-tetramethylguanidine).

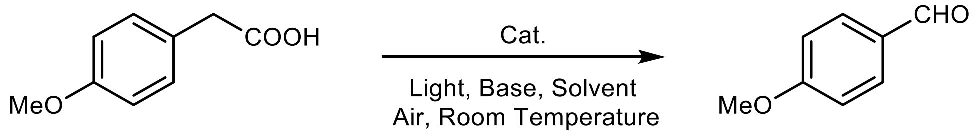

Entry	Light	Air	Catalyst	Time (h)	Solvent	Base	Additive	Conv. (%)
1	-	+	TFPA-TAPT-COF-Q	9	MeCN	TMG	-	0
2	+	-	TFPA-TAPT-COF-Q	9	MeCN	TMG	-	0
3	+	+	-	9	MeCN	TMG	-	0
4	+	+	TFPA-TAPT-COF-Q	7	MeCN	TMG	-	83
5	+	+	TFPA-TAPT-COF-Q	8	MeCN	TMG	-	93
6	+	+	TFPA-TAPT-COF-Q	9	MeCN	TMG	-	100 [85]
7	+	+	TFPA-TAPT-COF-Q	10	MeCN	TMG	-	100 [89]
8	+	+	TFPA-TAPT-COF-Q	9	MeOH	TMG	-	14
9	+	+	TFPA-TAPT-COF-Q	9	$CHCl_3$	TMG	-	15
10	+	+	TFPA-TAPT-COF-Q	9	THF	TMG	-	**N.D.**
11	+	+	TFPA-TAPT-COF-Q	9	DMF	TMG	-	13
12	+	+	TFPA-TAPT-COF-Q	9	MeCN	$CsCO_3$	-	62
13	+	+	TFPA-TAPT-COF-Q	9	MeCN	K_2CO_3	-	69
14	+	+	TFPA-TAPT-COF-Q	9	MeCN	KOH	-	80
15	+	+	TFPA-TAPT-COF-Q	9	MeCN	Et_3N	-	40
16	+	+	TFPA-TAPT-COF	9	MeCN	TMG	-	85 [76]
17	+	+	TFPA-TPB-COF-Q	9	MeCN	TMG	-	79 [91]
18	+	+	TFPA-TPB-COF	9	MeCN	TMG	-	85 [76]
19	+	+	TFPA-TAPT-COF-Q	9	MeCN	TMG	KI	**N.D.**
20	+	+	TFPA-TAPT-COF-Q	9	MeCN	TMG	BQ	62 [36]
21	+	+	TFPA-TAPT-COF-Q	9	MeCN	TMG	DABCO	82 [83]
22	+	+	TFPA-TAPT-COF-Q	9	$MeCN/H_2O$ (4:1)	TMG	-	100 [12]
23	+	O_2	TFPA-TAPT-COF-Q	9	MeCN	TMG	-	100 [97]

N.D.: The signal of the product was not detected. [a]Selectivity under the related condition.

Supplementary Table 6: Photocatalytic activity of TFPA-TAPT-COF-Q in the coupling of benzylamine.

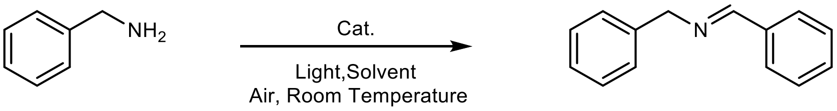

Entry	Light	Air	Catalyst	Time (h)	Solvent	Additive	Conv. (%)
1	-	+	TFPA-TAPT-COF-Q	3.5	MeCN	-	0
2	+	-	TFPA-TAPT-COF-Q	3.5	MeCN	-	0
3	+	+	-	3.5	MeCN	-	0
4	+	+	TFPA-TAPT-COF-Q	2	MeCN	-	83
5	+	+	TFPA-TAPT-COF-Q	3	MeCN	-	98
6	+	+	TFPA-TAPT-COF-Q	3.5	MeCN	-	100
7	+	+	TFPA-TAPT-COF-Q	4	MeCN	-	100
8	+	+	TFPA-TAPT-COF-Q	3.5	MeOH	-	12
9	+	+	TFPA-TAPT-COF-Q	3.5	$CHCl_3$	-	11
10	+	+	TFPA-TAPT-COF-Q	3.5	THF	-	68
11	+	+	TFPA-TAPT-COF	3.5	MeCN	-	100
12	+	+	TFPA-TPB-COF-Q	3.5	MeCN	-	80
13	+	+	TFPA-TPB-COF	3.5	MeCN	-	42
14	+	+	TFPA-TAPT-COF-Q	3.5	MeCN	BQ	**N.D.**
15	+	+	TFPA-TAPT-COF-Q	3.5	MeCN	DABCO	66
16	+	+	TFPA-TAPT-COF-Q	3.5	MeCN	KI	19

N.D.: The signal of the products was not detected.

Supplementary Table 7: Comparison of COF-based photocatalysts for photocatalytic H_2O_2 production yield in different conditions.

Photocatalyst	H_2O_2 yield (μmol g^{-1} h^{-1})	Irradiated conditions	Solvents	Ref.
TFPA-TAPT-COF-Q	11831.6	full-wave band	H_2O: BA (10: 1)	This work
TFPA-TPB-COF-Q	9251.0	full-wave band	H_2O: BA (10: 1)	
DMCR-1NH	2588	λ>420 nm	H_2O: IPA (10: 1)	12
PMCR-1	5500	λ>420 nm	H_2O: BA (10: 1)	14
COF-TpBPy	6946.7	λ>420 nm	H_2O	16
TZ-COF	4951	λ>420 nm	H_2O: BA (1: 1)	17
COF-TTA-TTTA	4347	LED (λ≈420 nm)	H_2O: EtOH (9: 1)	18

Supplementary Figures:

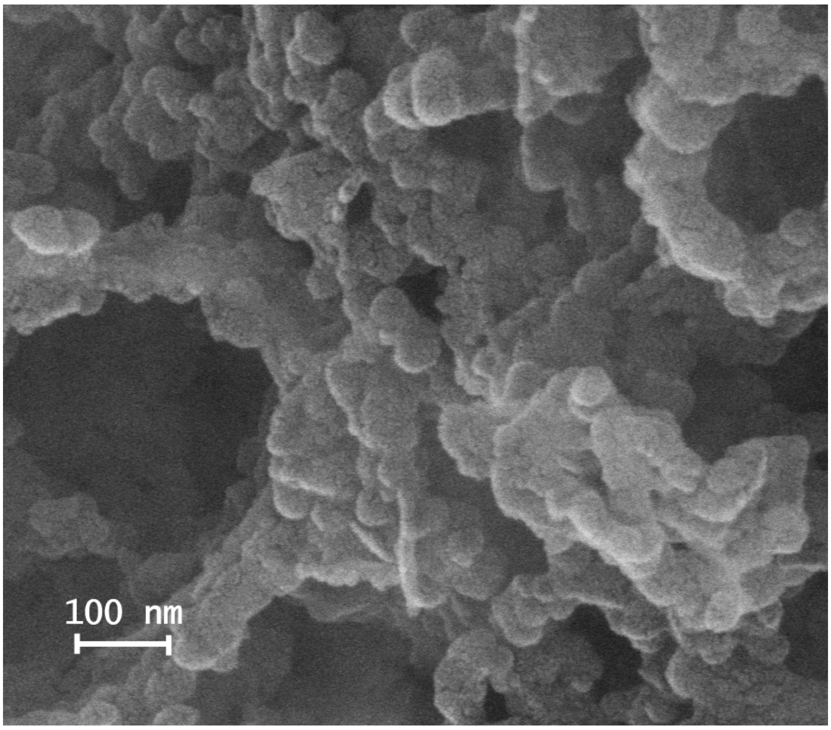

Supplementary Figure 1. SEM image of TFPA-TAPT-COF-Q.

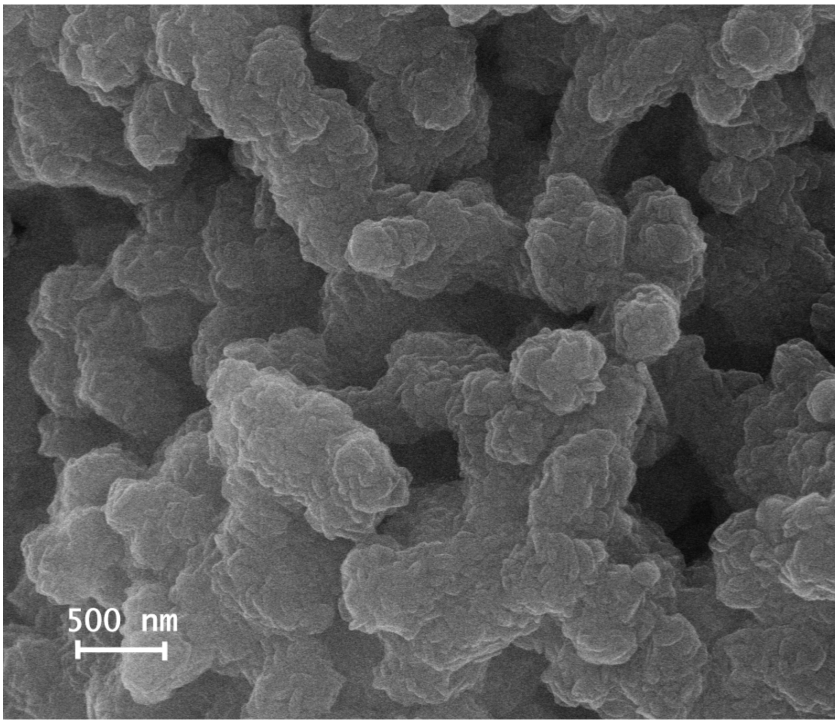

Supplementary Figure 2. SEM image of TFPA-TAPT-COF.

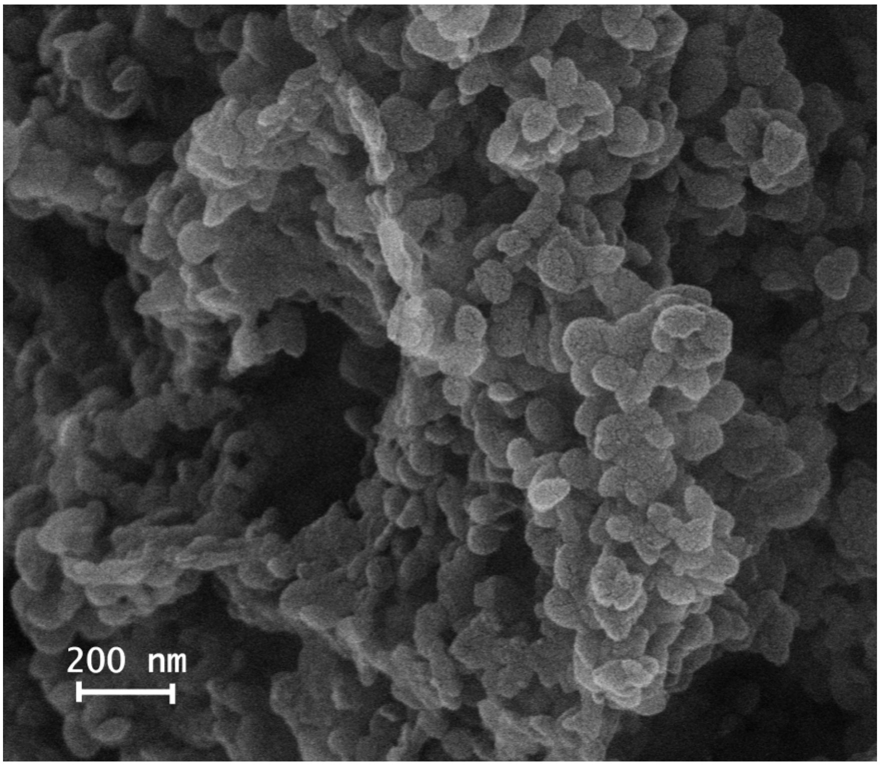

Supplementary Figure 3. SEM image of TFPA-TPB-COF-Q.

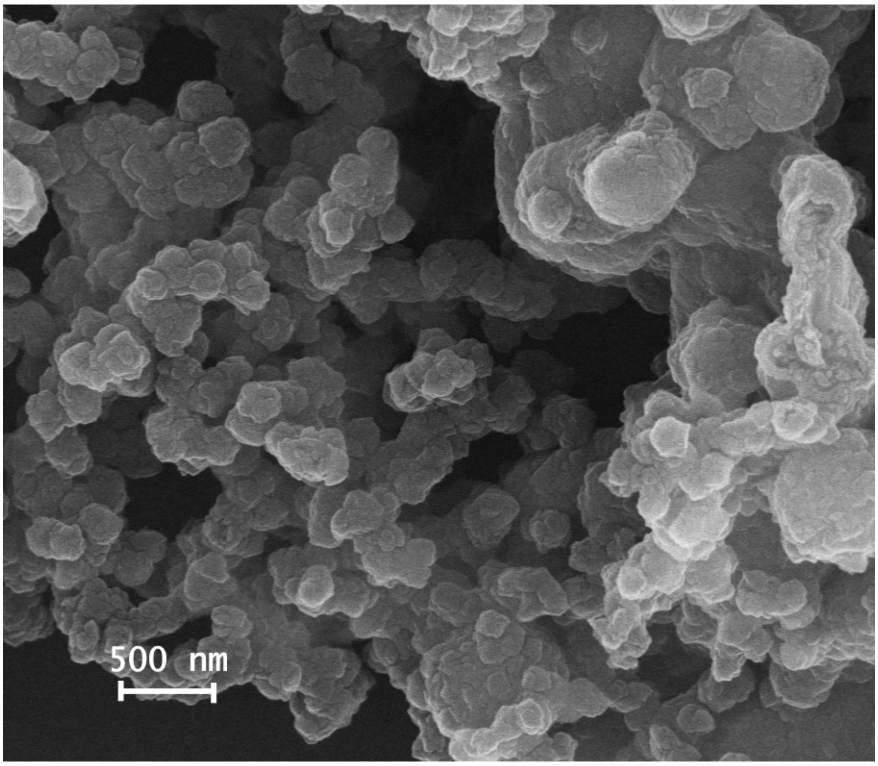

Supplementary Figure 4. SEM image of TFPA-TPB-COF.

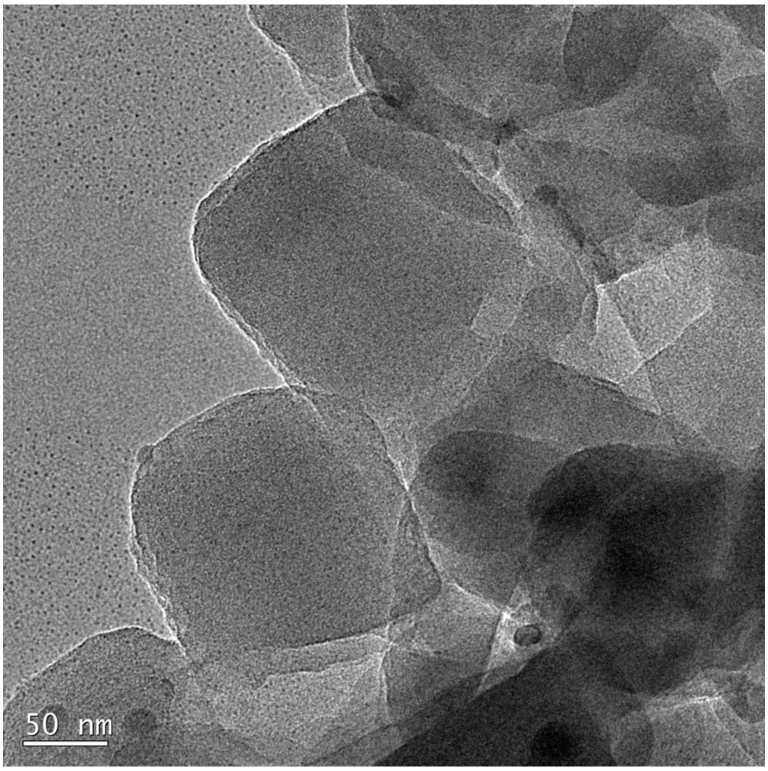

Supplementary Figure 5. TEM image of TFPA-TAPT-COF.

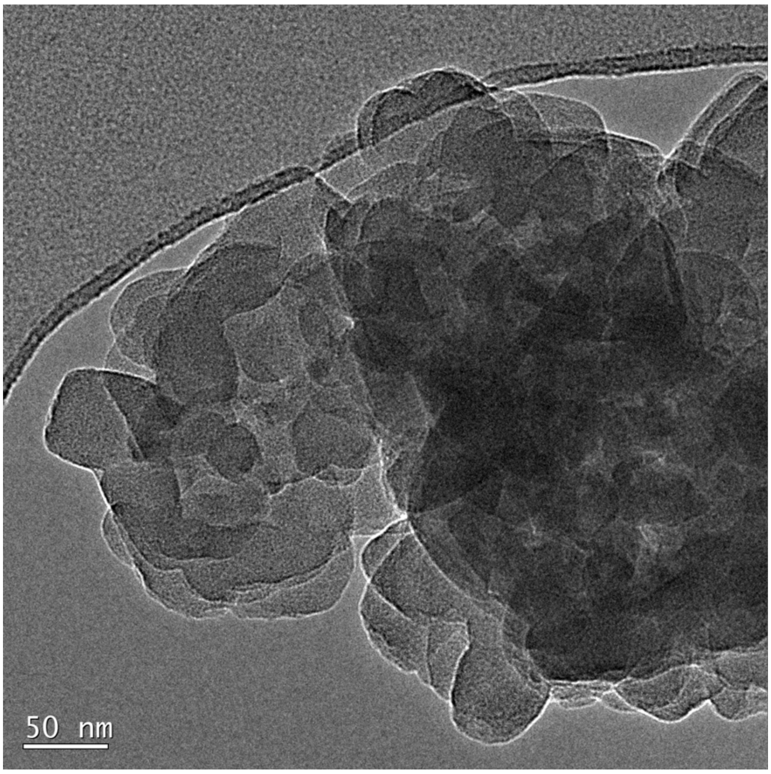

Supplementary Figure 6. TEM image of TFPA-TPB-COF.

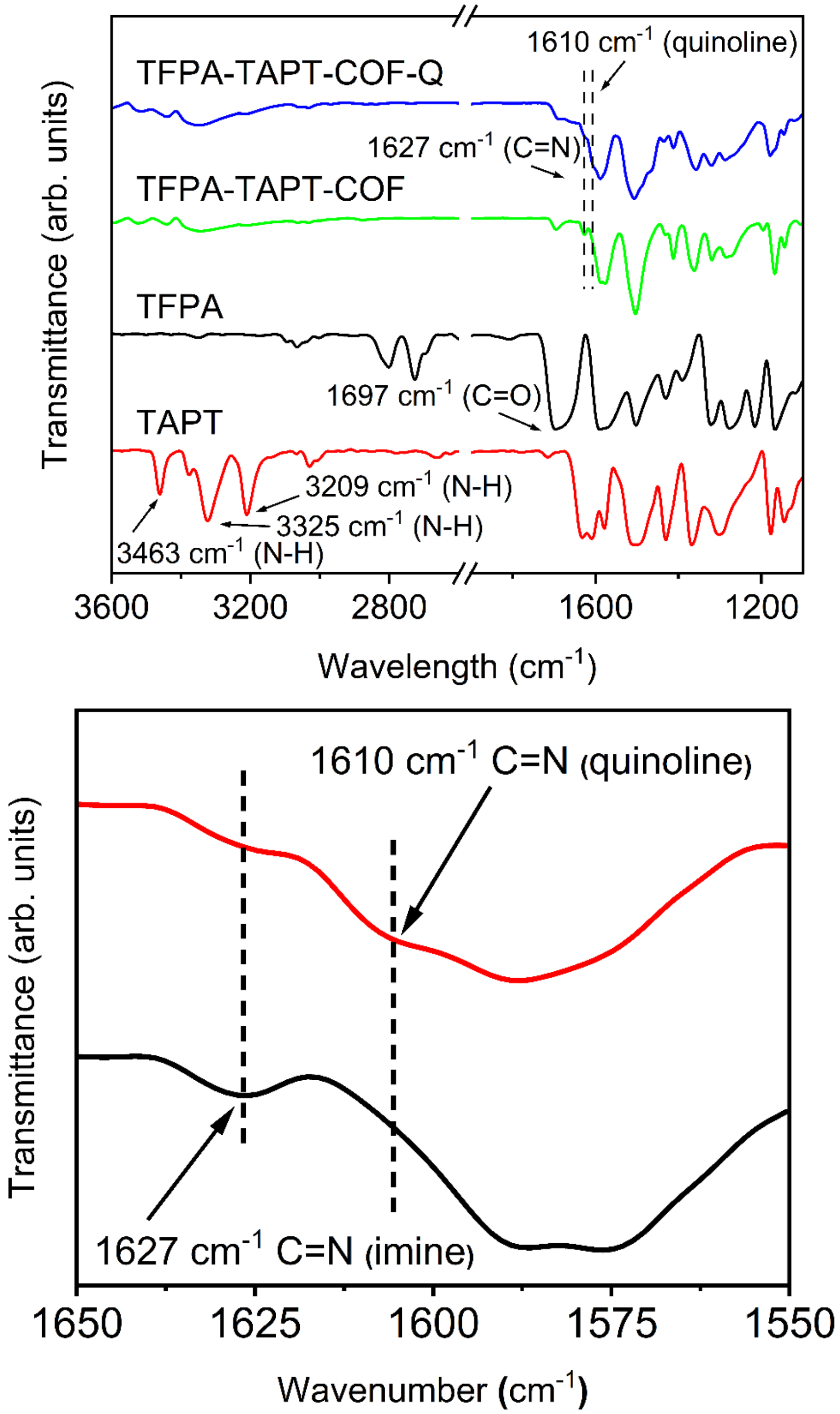

Supplementary Figure 7. **FT-IR spectra.** FT-IR spectra (up) of TFPA-TAPT-COF-Q (blue), TFPA-TAPT-COF (green) and their monomers (black line for TFPA and red line for TAPT) and locally enlarged spectra (down) of TFPA-TAPT-COF-Q (red) and TFPA-TAPT-COF (black).

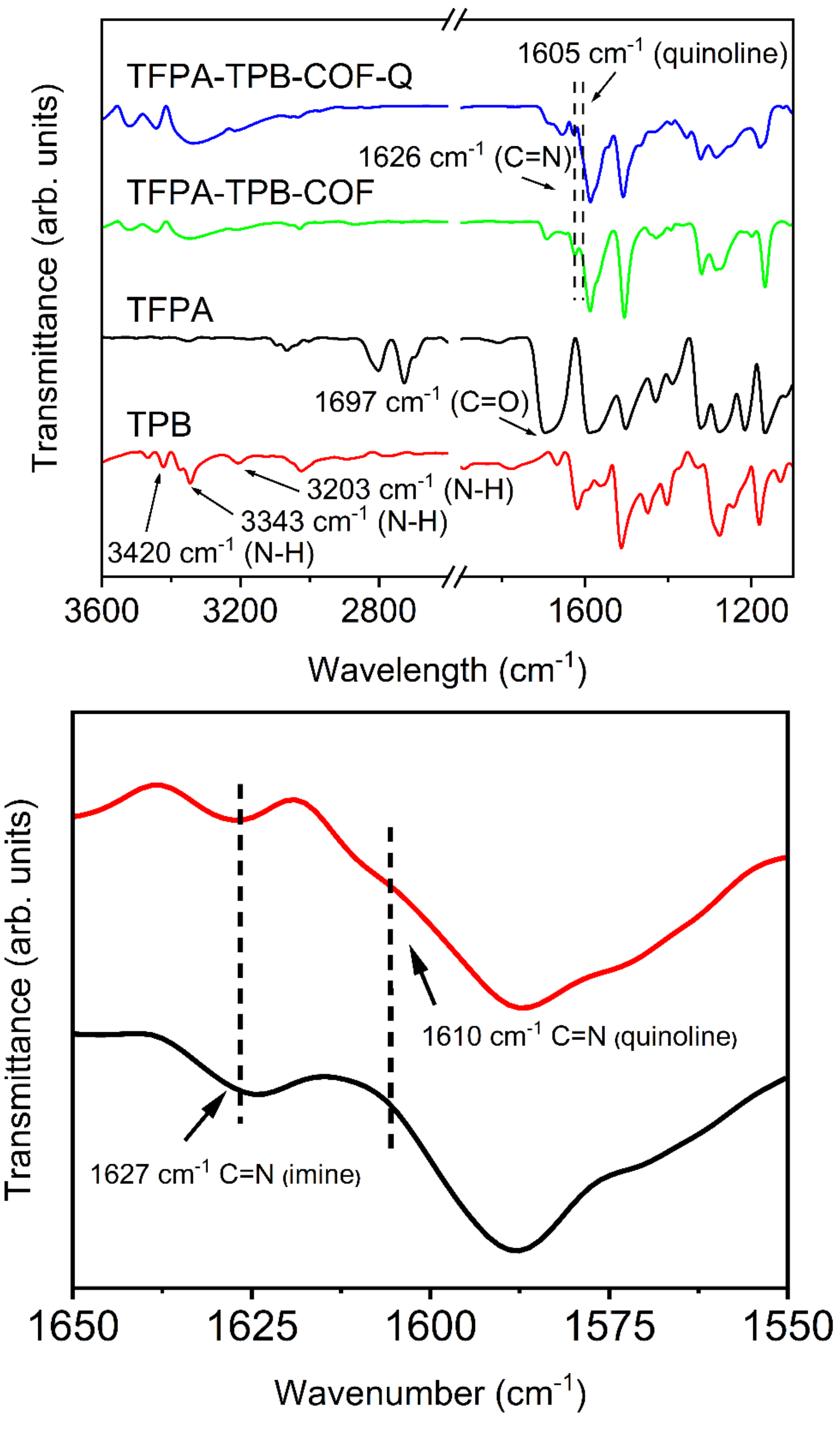

Supplementary Figure 8. **FT-IR spectra.** FT-IR spectra (up) of TFPA-TPB-COF-Q (blue), TFPA-TPB-COF (green) and their monomers (black line for TFPA and red line for TPB) and locally enlarged spectra (down) of TFPA-TPB-COF-Q (red) and TFPA-TPB-COF (black).

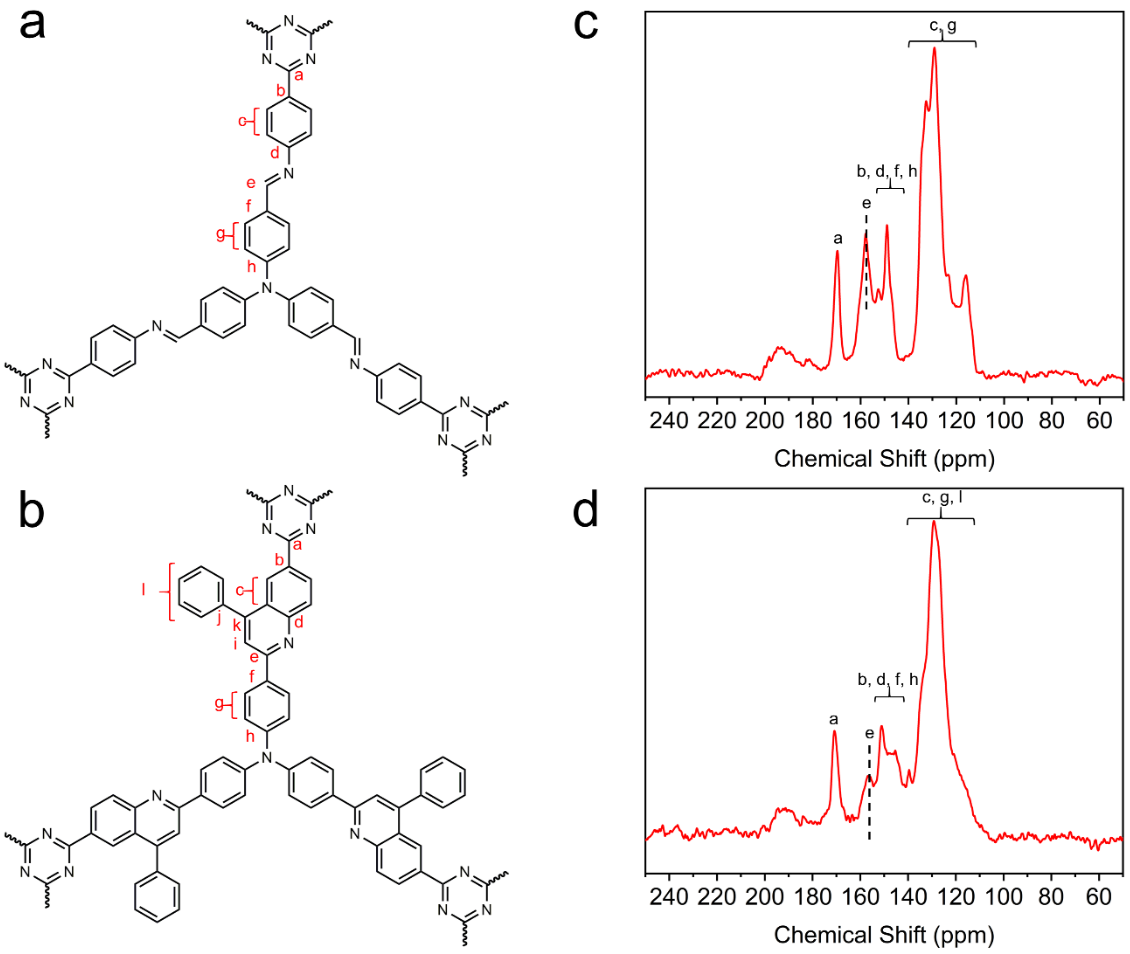

Supplementary Figure 9. **Chemical structures and solid-sate ^{13}C NMR spectra.** Chemical structures of a) TFPA-TAPT-COF and b) TFPA-TAPT-COF-Q, and related solid-sate ^{13}C NMR spectra of c) TFPA-TAPT-COF and d) TFPA-TAPT-COF-Q.

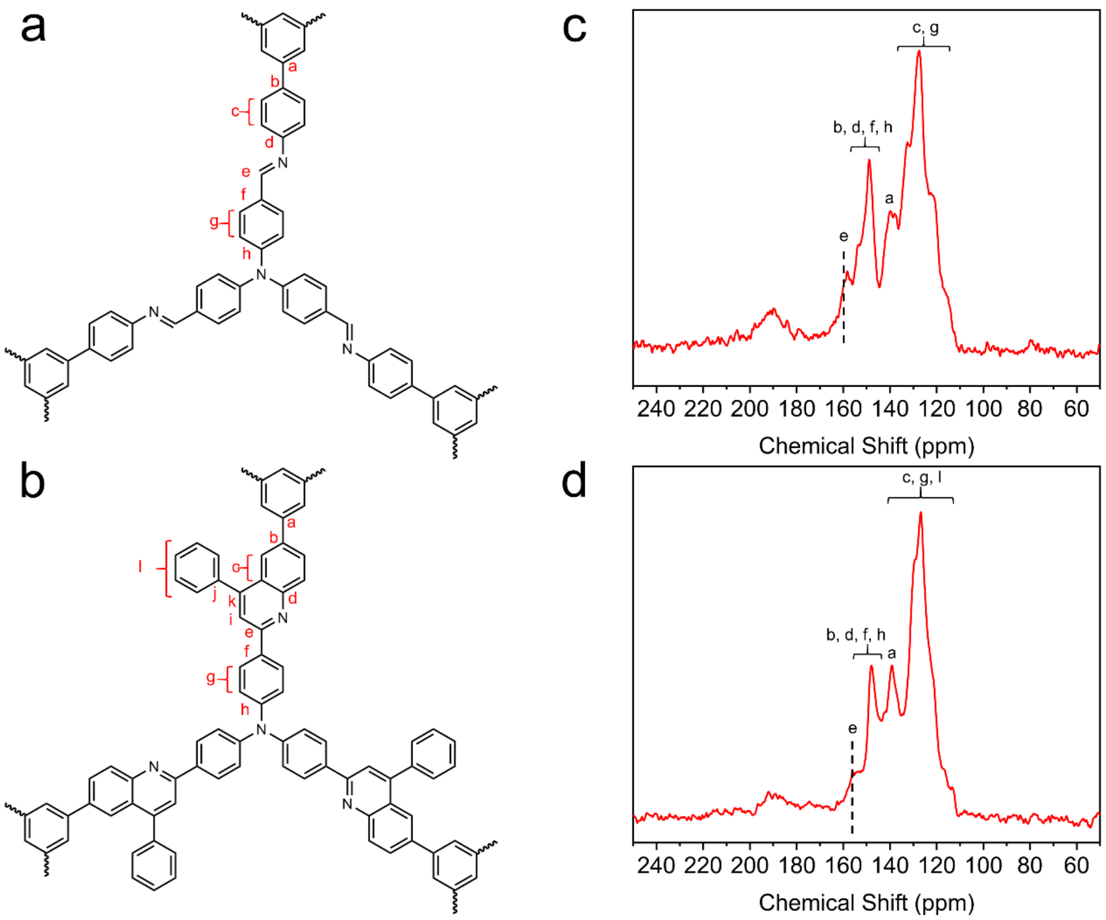

Supplementary Figure 10. **Chemical structures and solid-sate ^{13}C NMR spectra.** Chemical structures of a) TFPA-TPB-COF and b) TFPA-TPB-COF-Q, and related solid-sate ^{13}C NMR spectra of c) TFPA-TPB-COF and d) TFPA-TPB-COF-Q.

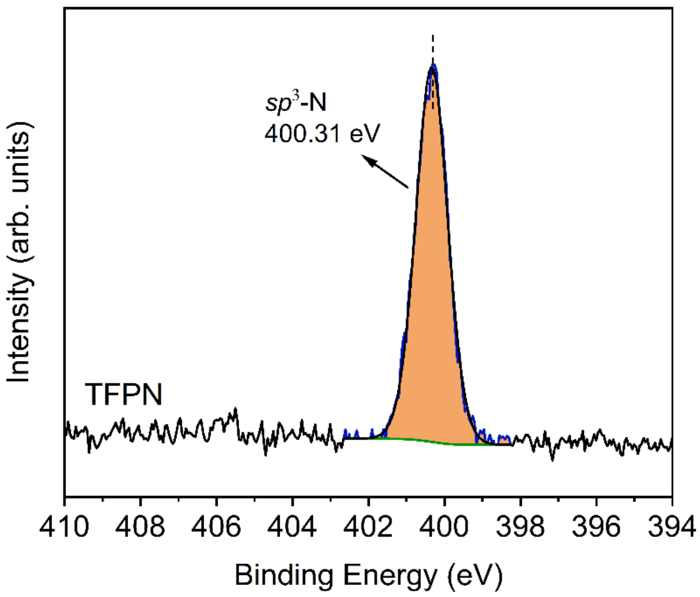

Supplementary Figure 11. XPS spectrum of N 1*s* in TFPA monomer.

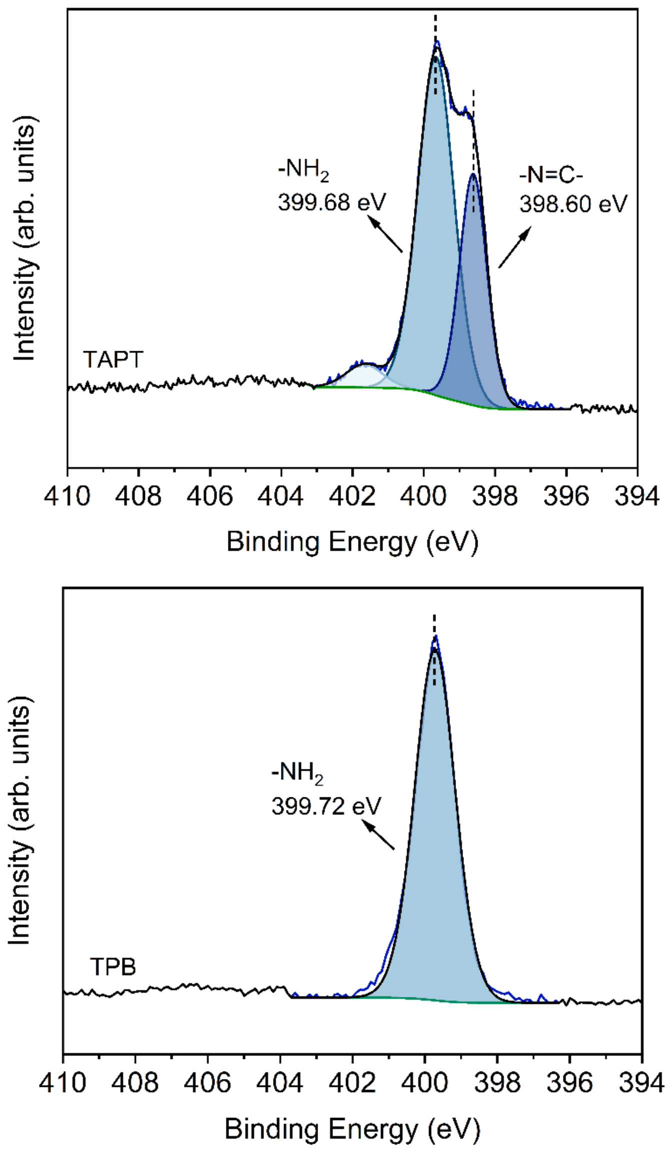

Supplementary Figure 12. **XPS spectra.** XPS spectra of N 1*s* in TAPT monomer (up) and TPB monomer (down).

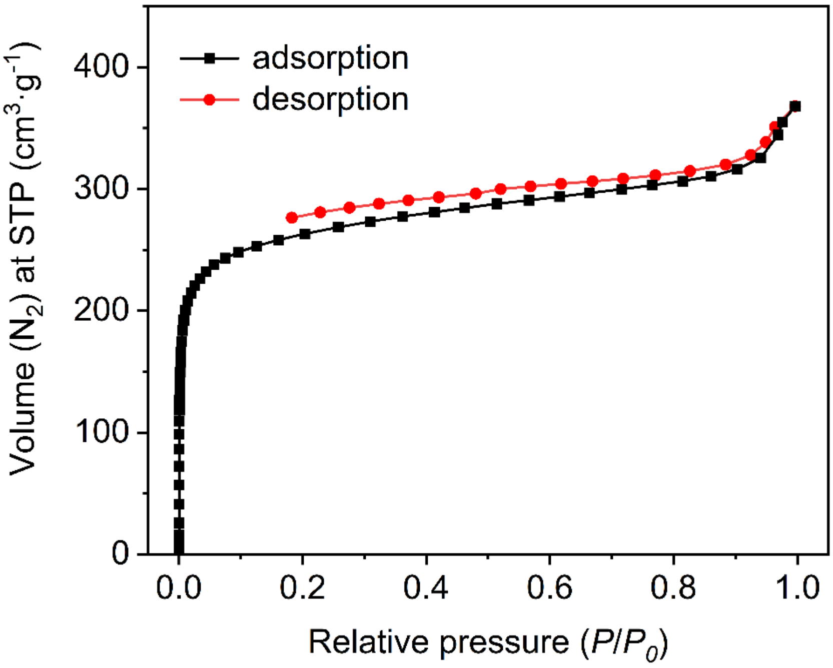

Supplementary Figure 13. N_2 sorption isotherm (77 K) of TFPA-TAPT-COF.

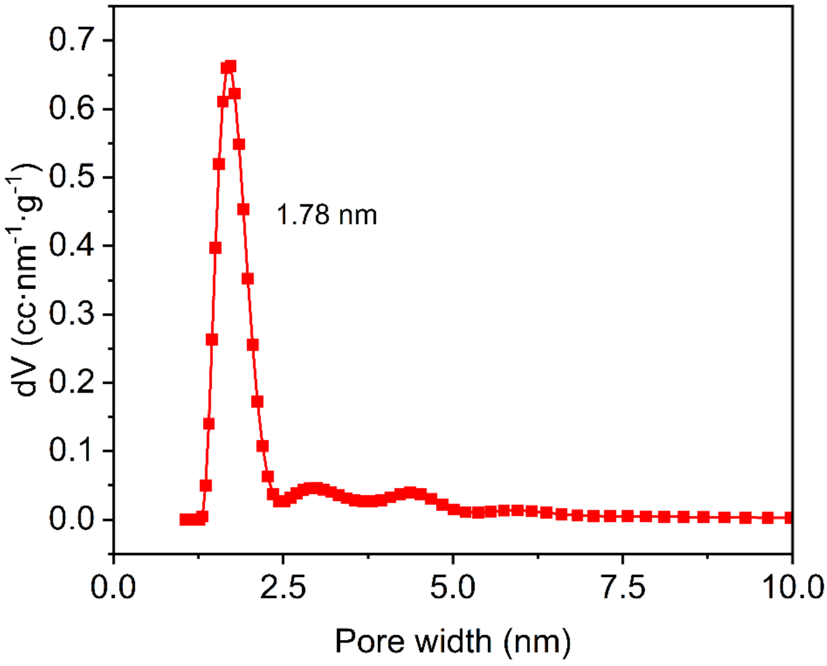

Supplementary Figure 14. Pore size distribution of TFPA-TAPT-COF.

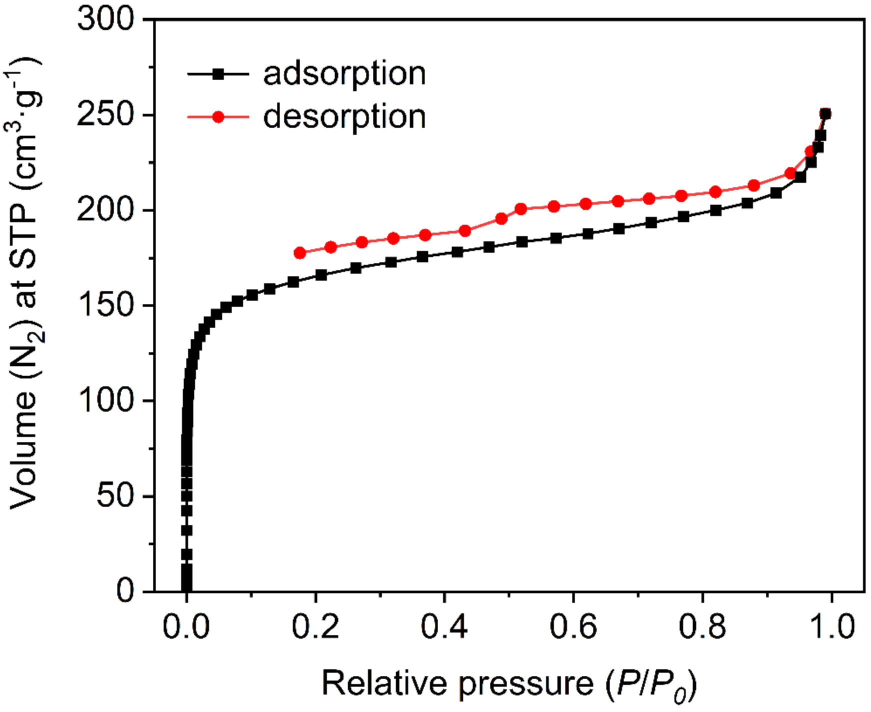

Supplementary Figure 15. N_2 sorption isotherm (77 K) of TFPA-TPB-COF.

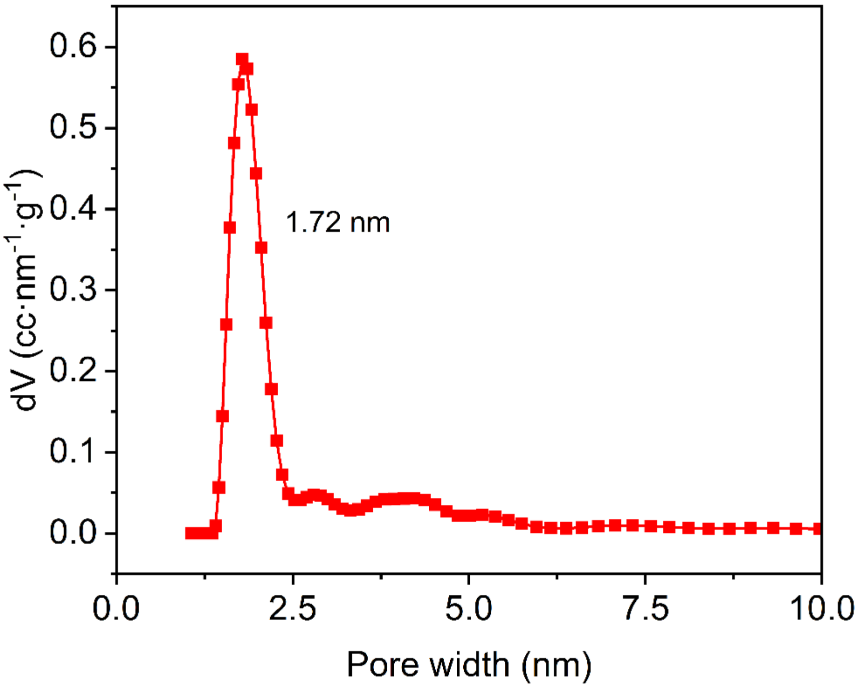

Supplementary Figure 16. Pore size distribution of TFPA-TPB-COF.

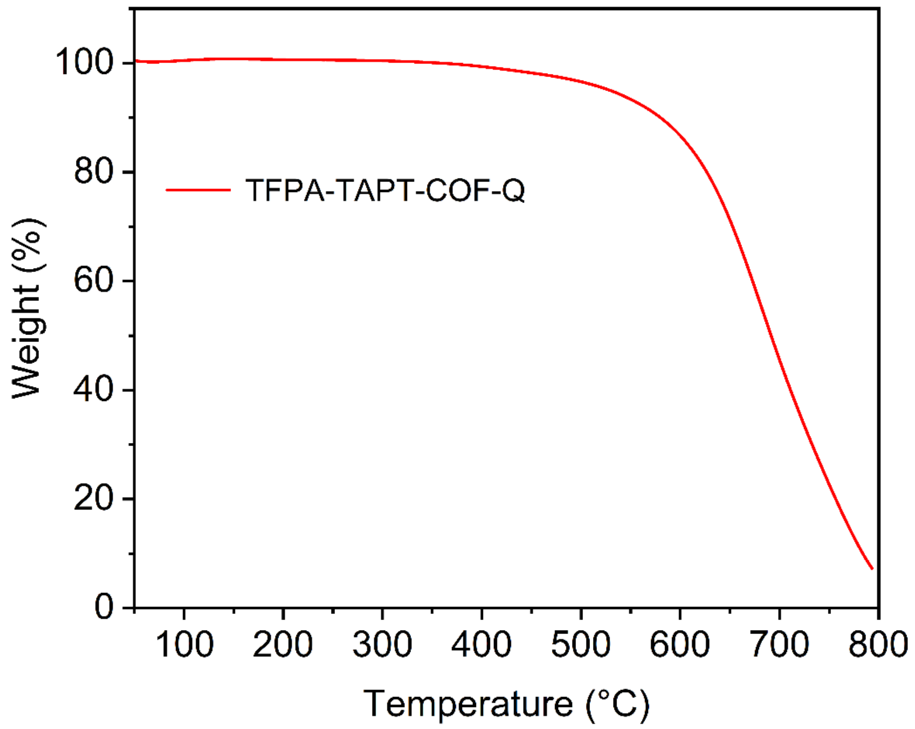

Supplementary Figure 17. TGA curve of TFPA-TAPT-COF-Q.

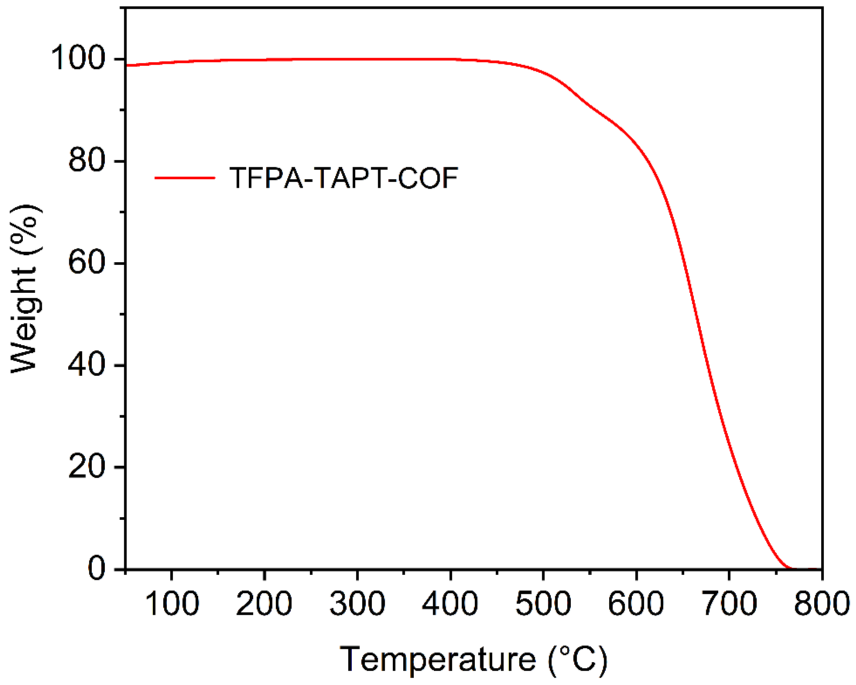

Supplementary Figure 18. TGA curve of TFPA-TAPT-COF.

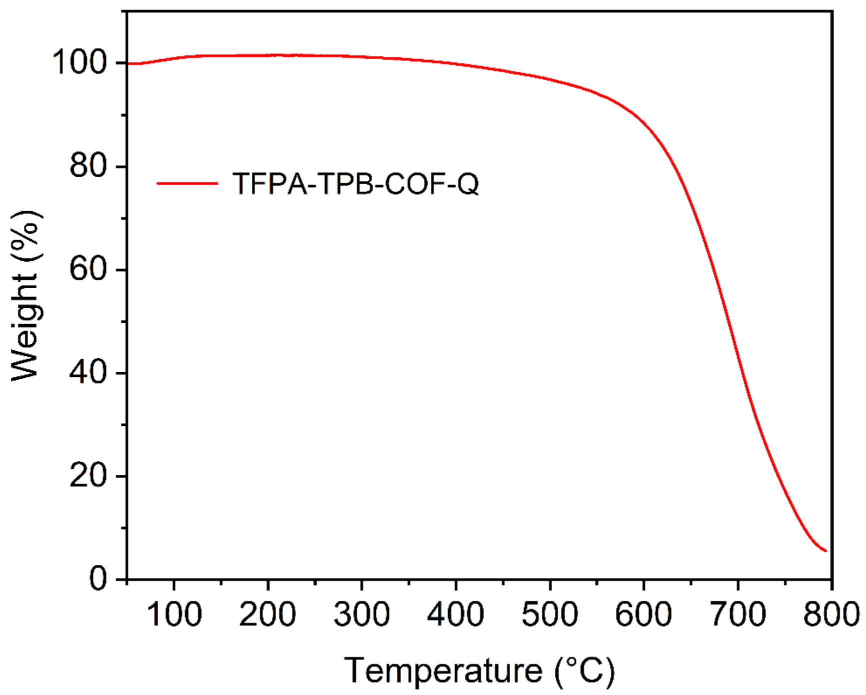

Supplementary Figure 19. TGA curve of TFPA-TPB-COF-Q.

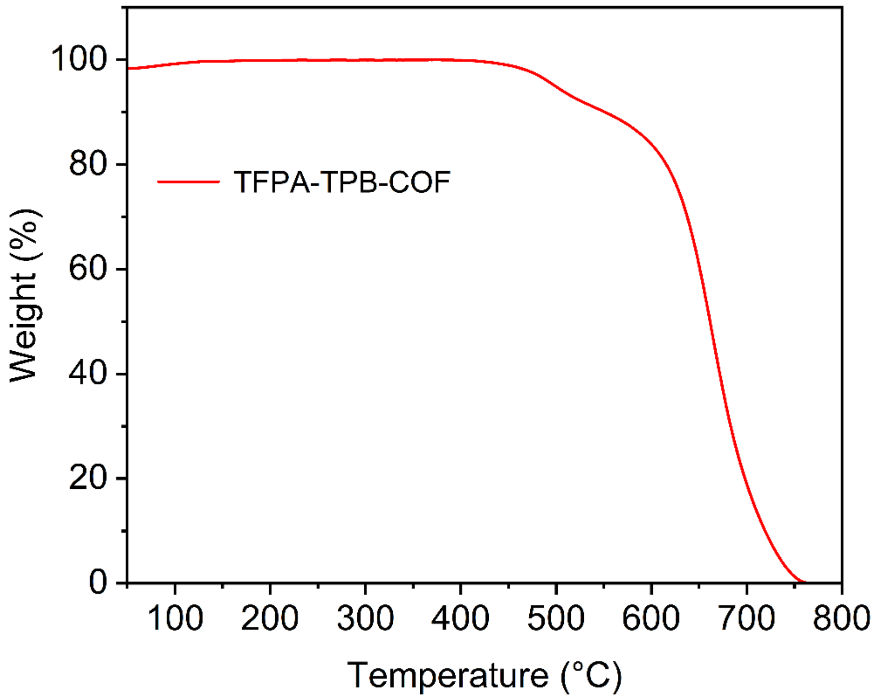

Supplementary Figure 20. TGA curve of TFPA-TPB-COF.

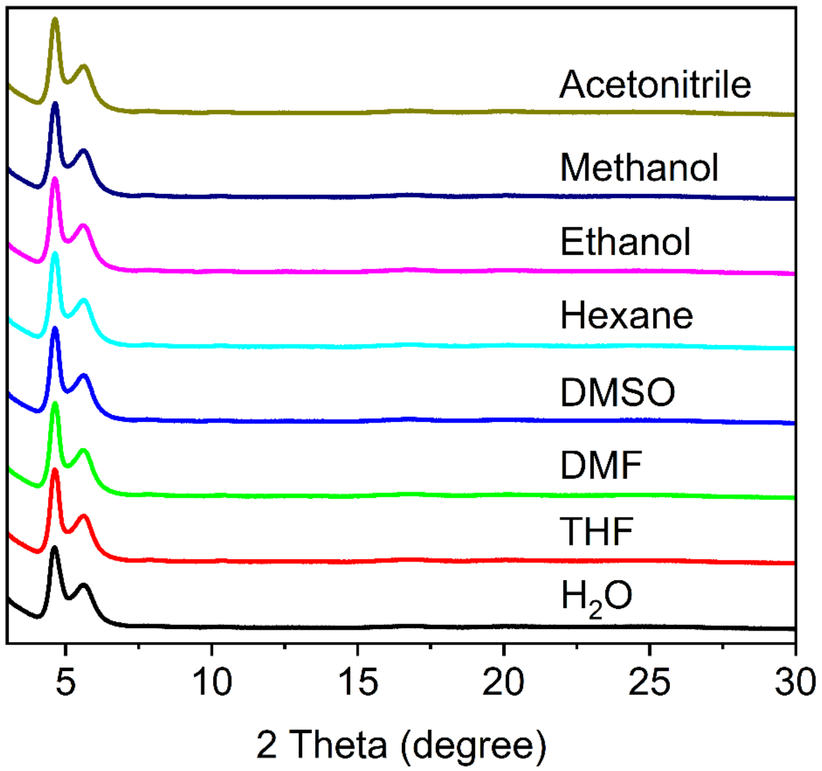

Supplementary Figure 21. PXRD patterns of TFPA-TAPT-COF in different solvents.

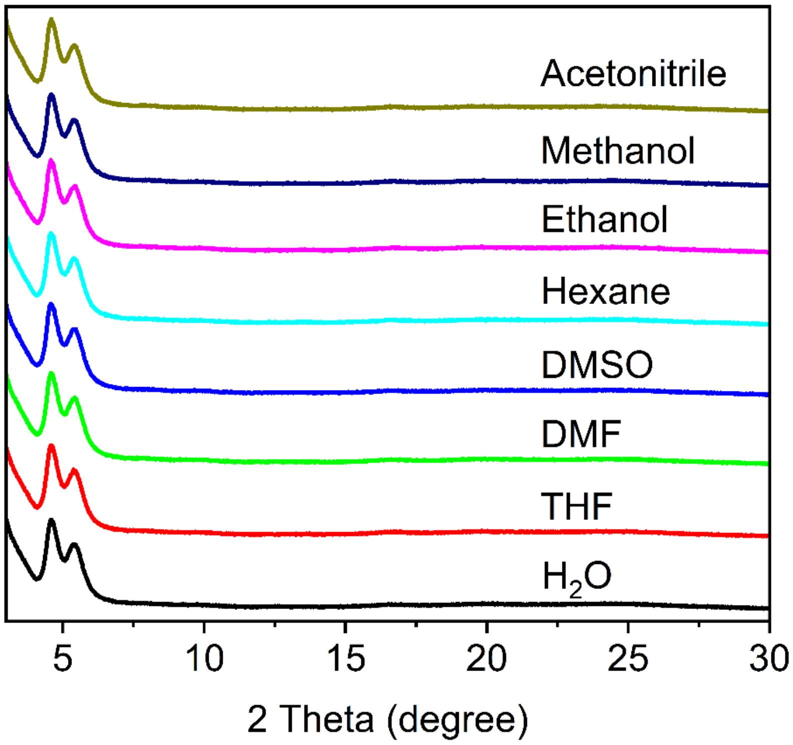

Supplementary Figure 22. PXRD patterns of TFPA-TAPT-COF-Q in different solvents.

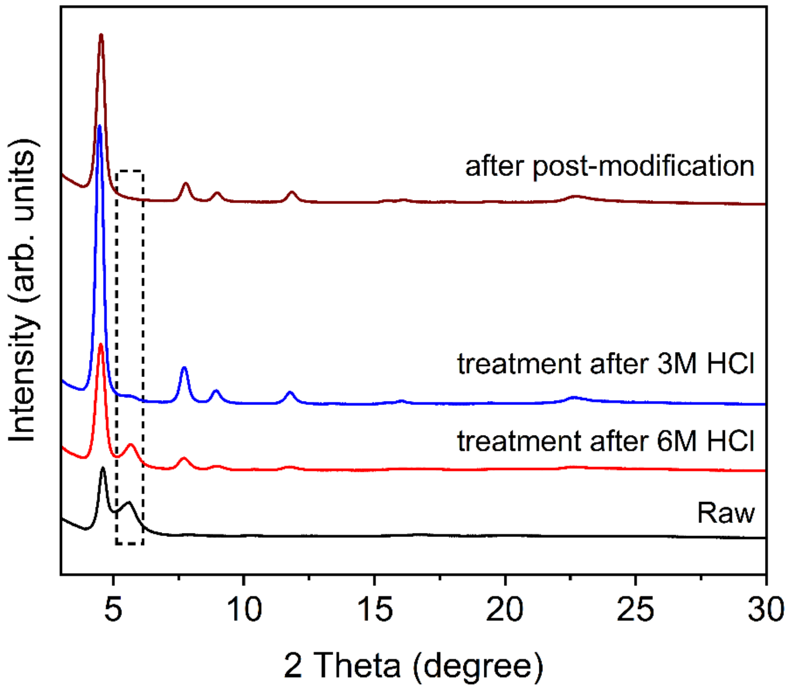

Supplementary Figure 23. PXRD patterns of TFPA-TAPT-COF (black), TFPA-TAPT-COF-Q (brown), and TFPA-TAPT-COF-H (the name for the samples of TFPA-TAPT-COF treated by HCl solution, blue for treatment by 3M HCl after 8 h and red for treatment by 6M HCl after 8 h).[19]

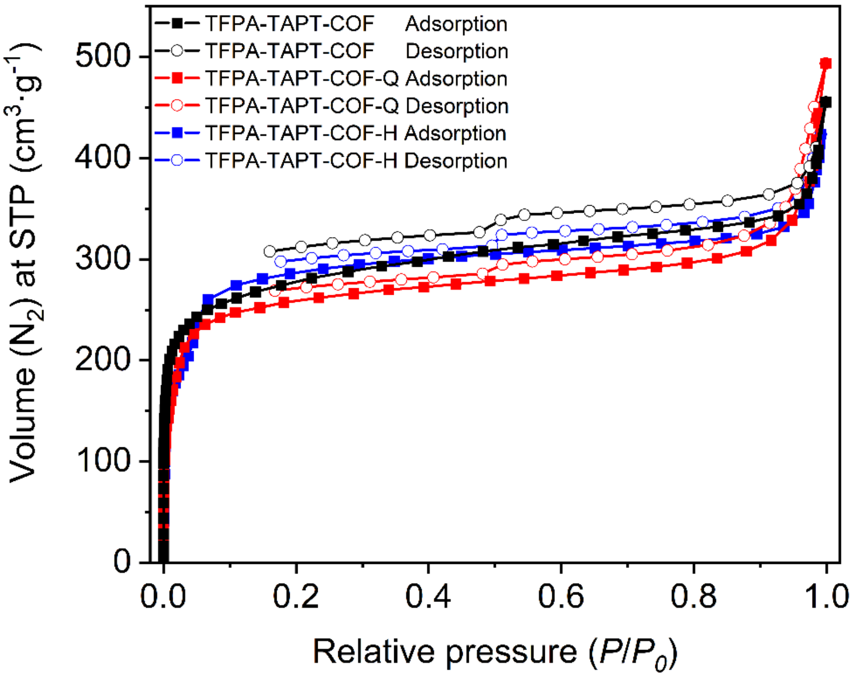

Supplementary Figure 24. N_2 sorption isotherm (77 K) of TFPA-TAPT-COF (black), TFPA-TAPT-COF-Q (red) and TFPA-TAPT-COF-H (blue).

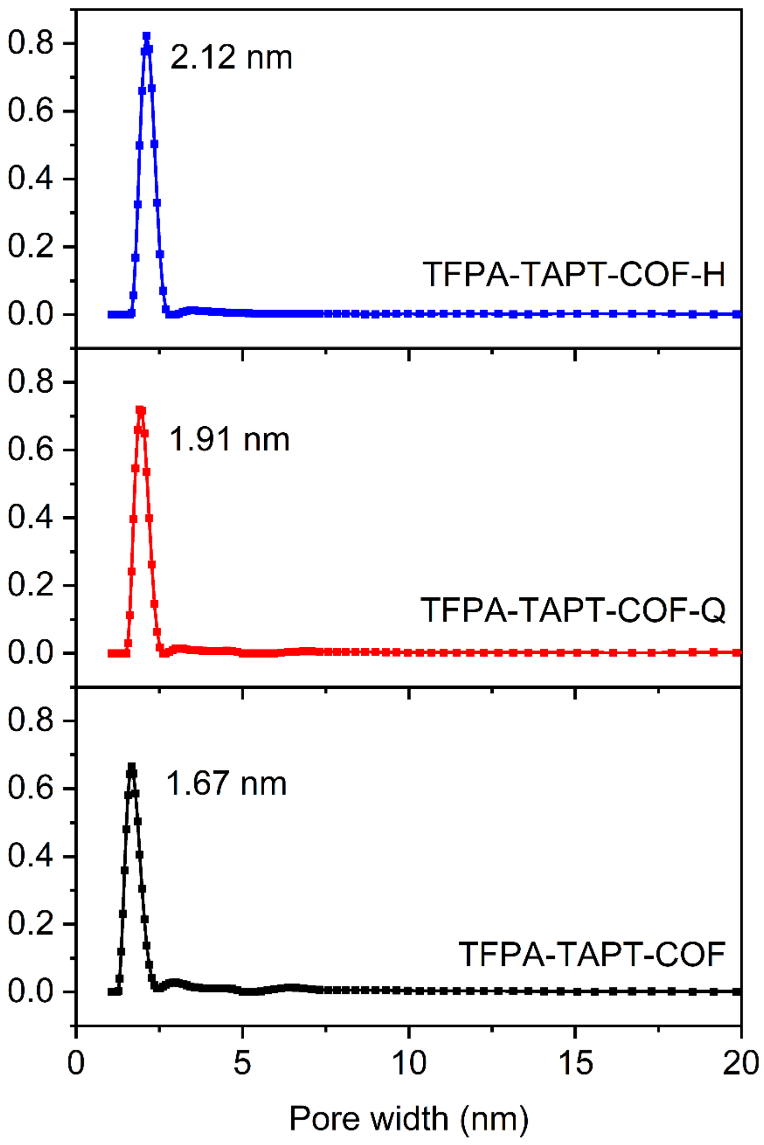

Supplementary Figure 25. Calculated pore size distributions of TFPA-TAPT-COF (black), TFPA-TAPT-COF-Q (red) and TFPA-TAPT-COF-H (blue).

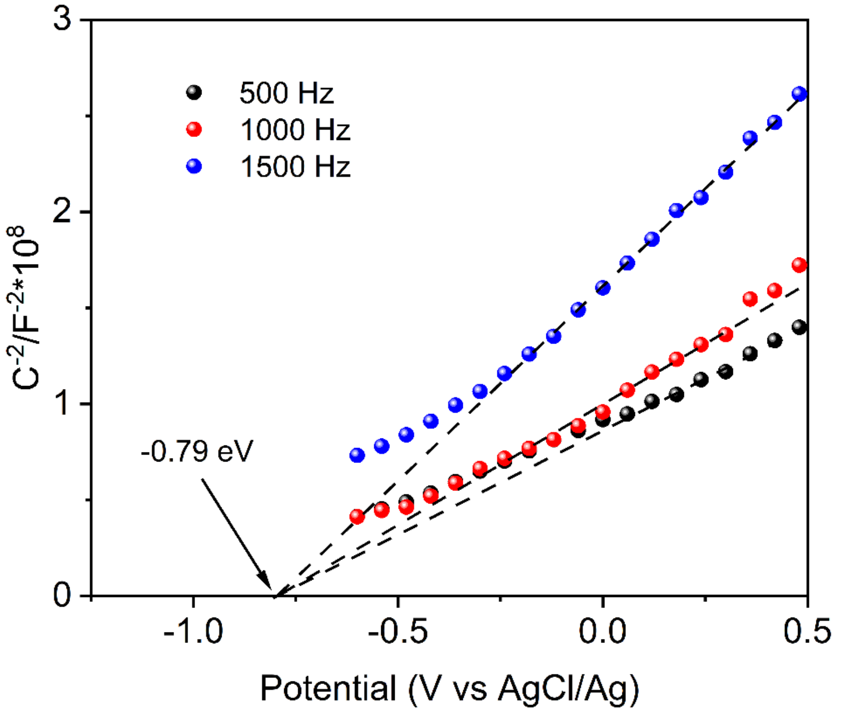

Supplementary Figure 26. Mott−Schottky plots of TFPA-TAPT-COF.

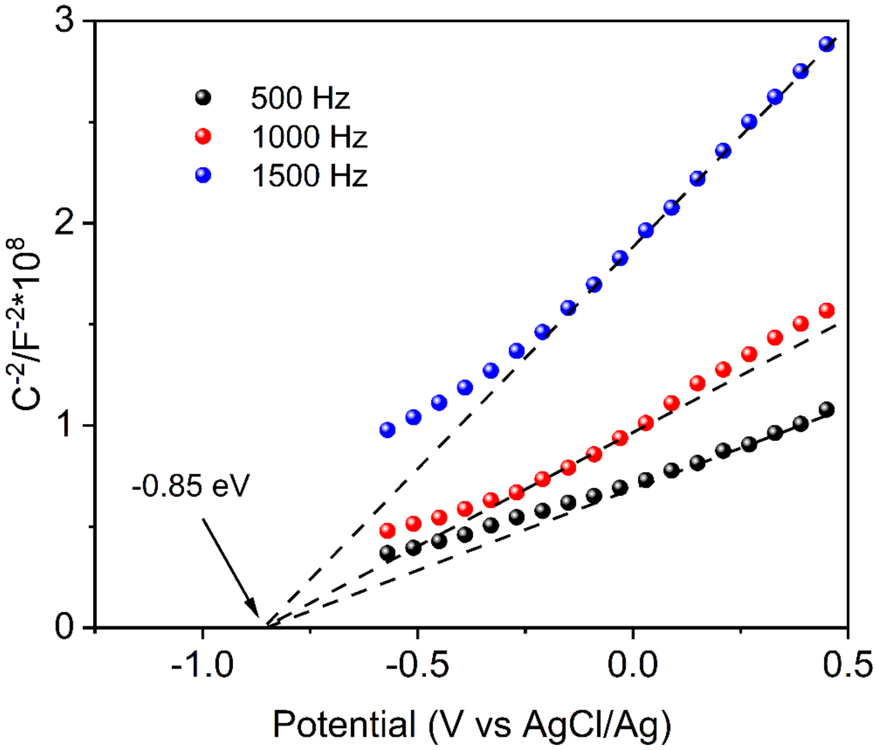

Supplementary Figure 27. Mott−Schottky plots of TFPA-TPB-COF.

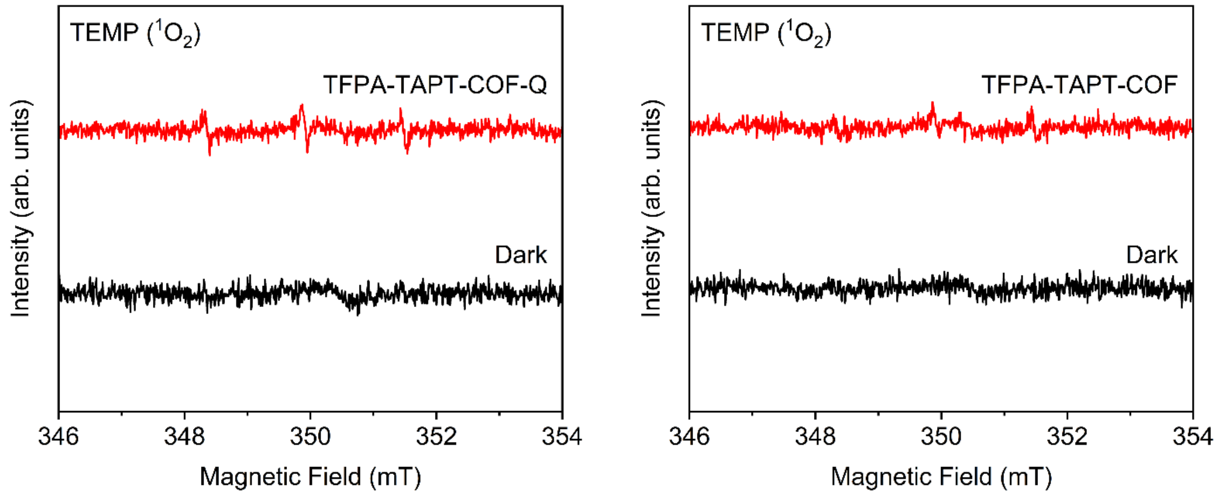

Supplementary Figure 28. **EPR spectra.** EPR spectra in MeCN under dark (black) and the irradiation of the Xe lamp with TEMP as the trapping agent for TFPA-TAPT-COF-Q and TFPA-TAPT-COF.

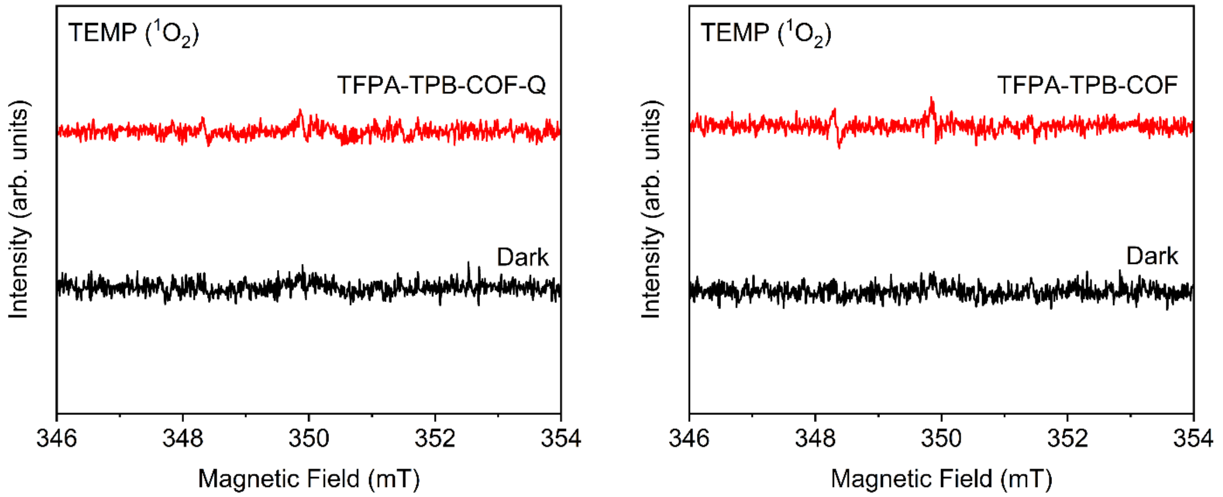

Supplementary Figure 29. **EPR spectra.** EPR spectra in MeCN under dark (black) and the irradiation of the Xe lamp with TEMP as the trapping agent for TFPA-TPB-COF-Q and TFPA-TPB-COF.

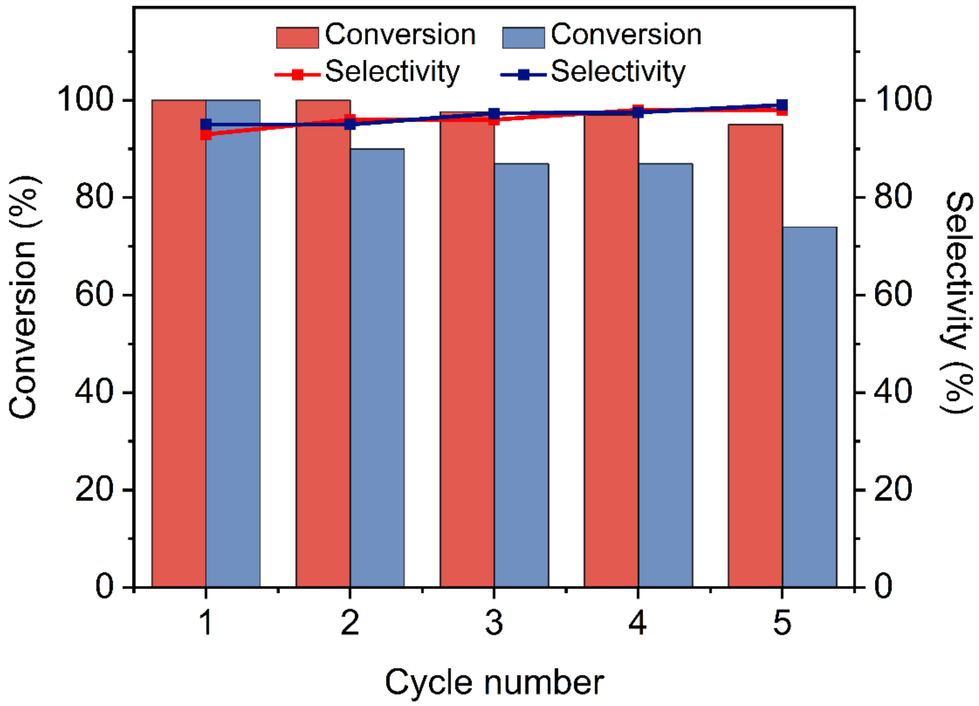

Supplementary Figure 30. Five runs of photocatalytic oxidation of sulfide with TFPA-TAPT-COF-Q (red) and TFPA-TAPT-COF (blue) as catalysts.

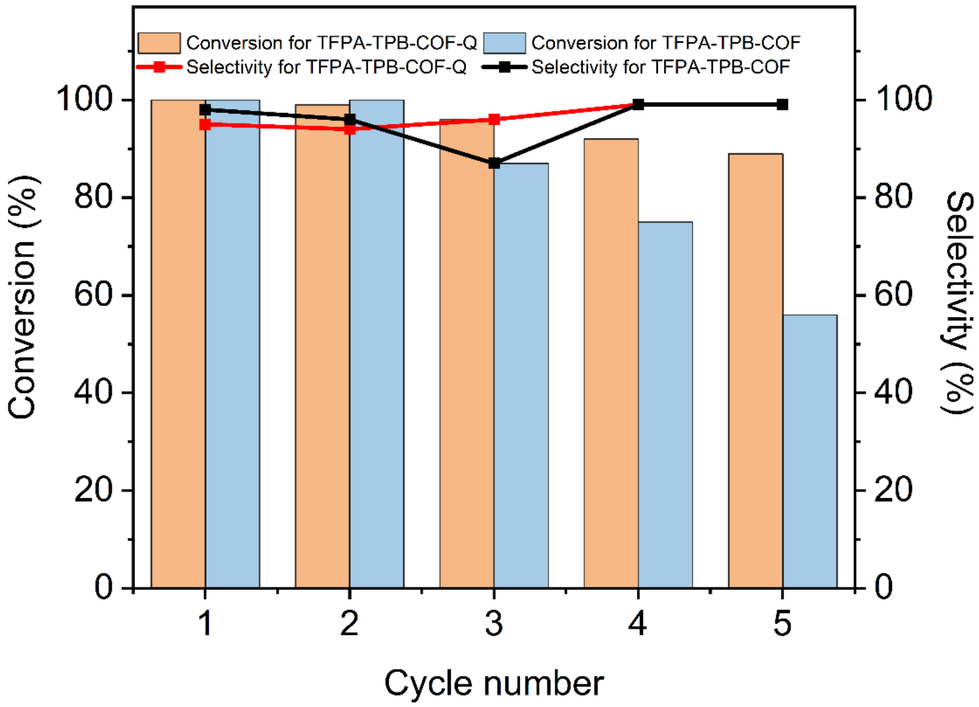

Supplementary Figure 31. Five runs of photocatalytic oxidation of sulfide with TFPA-TPB-COF-Q (orange) and TFPA-TPB-COF (light blue) as catalysts.

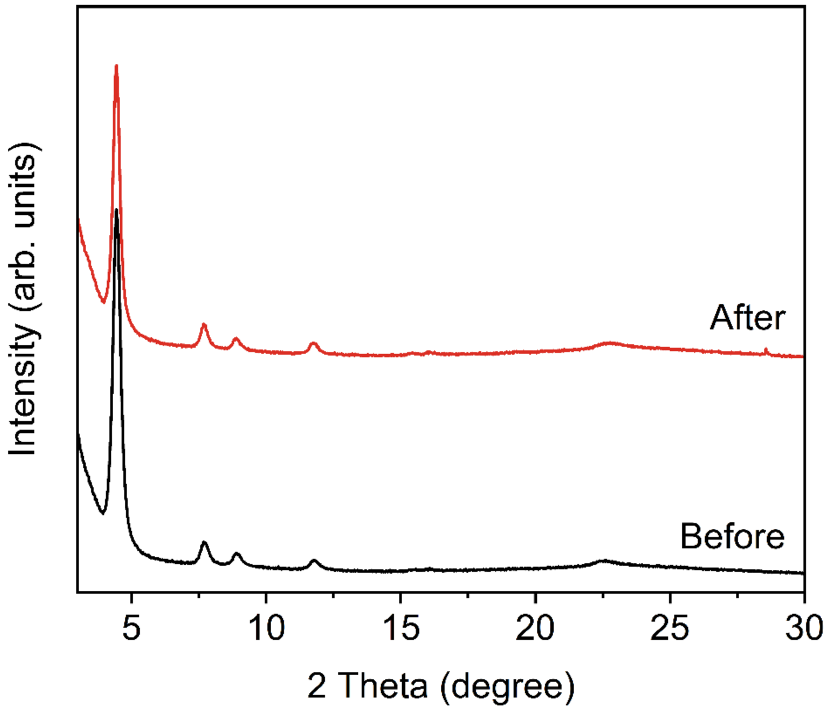

Supplementary Figure 32. PXRD patterns of TFPA-TAPT-COF-Q before and after five runs in oxidation of sulfide.

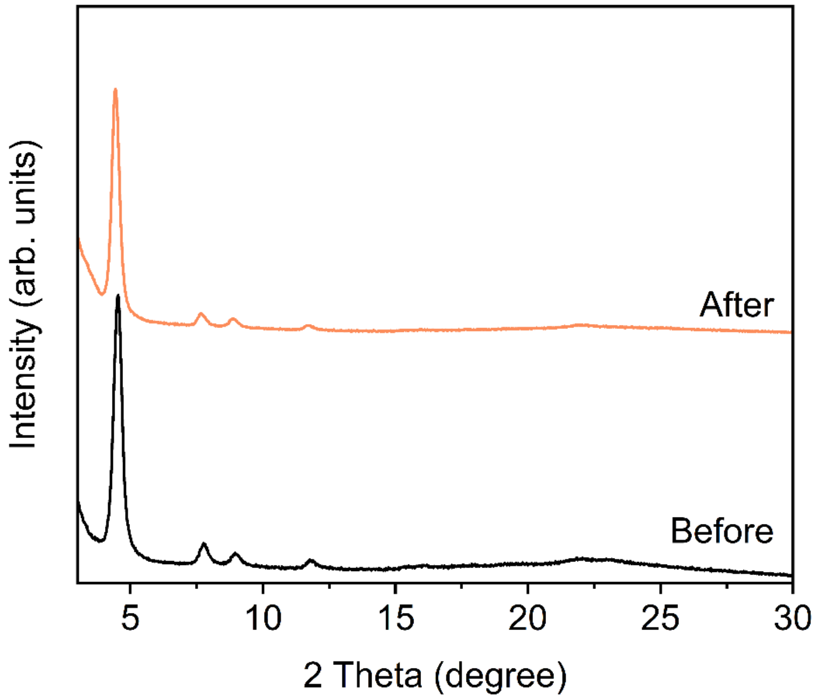

Supplementary Figure 33. PXRD patterns of TFPA-TPB-COF-Q before and after five runs in oxidation of sulfide.

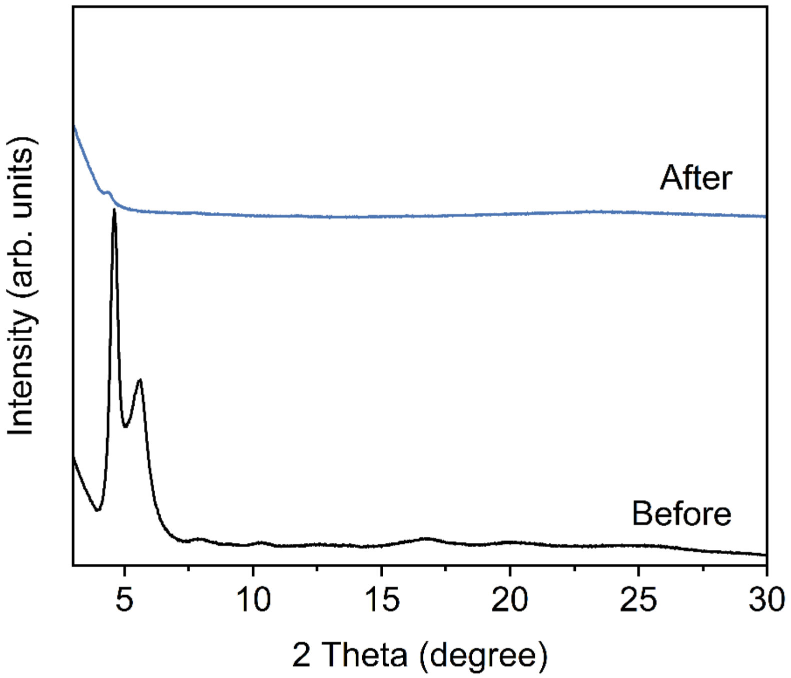

Supplementary Figure 34. PXRD patterns of TFPA-TAPT-COF before and after five runs of sulfide oxidation.

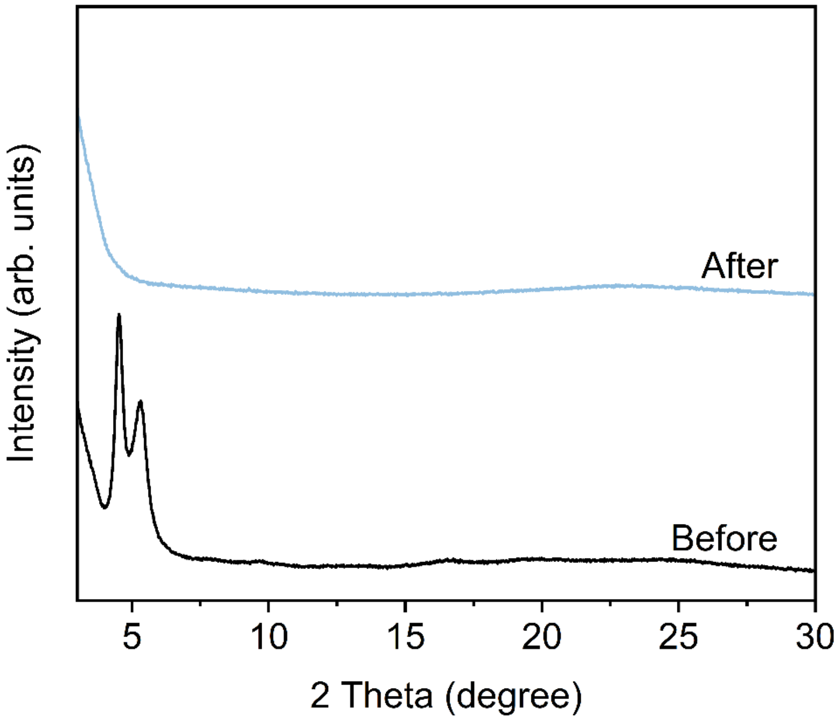

Supplementary Figure 35. PXRD patterns of TFPA-TPB-COF before and after five runs of sulfide oxidation.

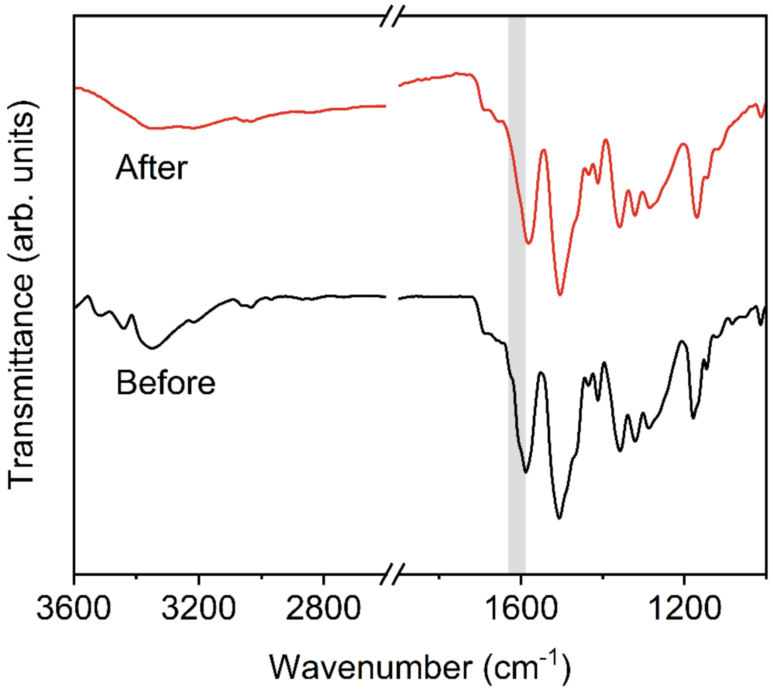

Supplementary Figure 36. FT-IR spectra of TFPA-TAPT-COF-Q before and after five runs of sulfide oxidation.

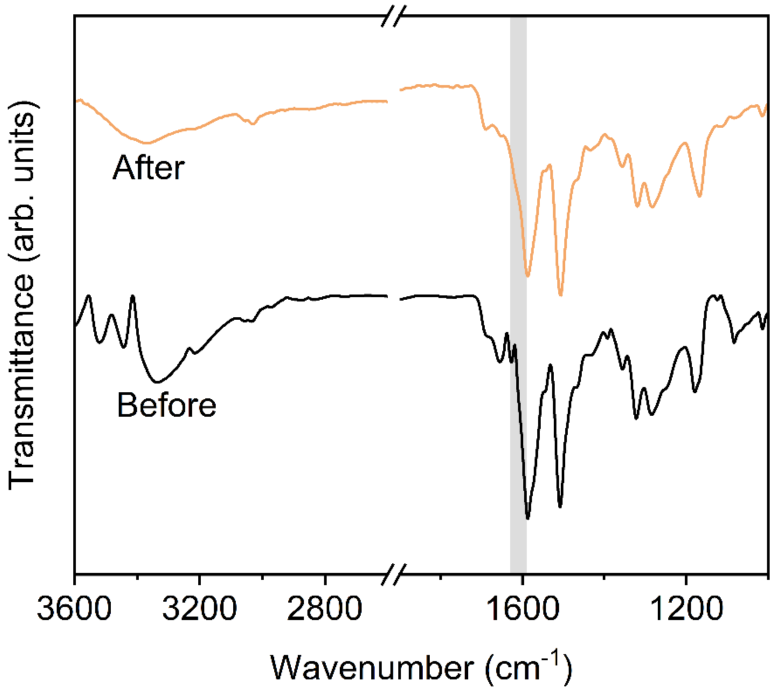

Supplementary Figure 37. FT-IR spectra of TFPA-TPB-COF-Q before and after five runs of sulfide oxidation.

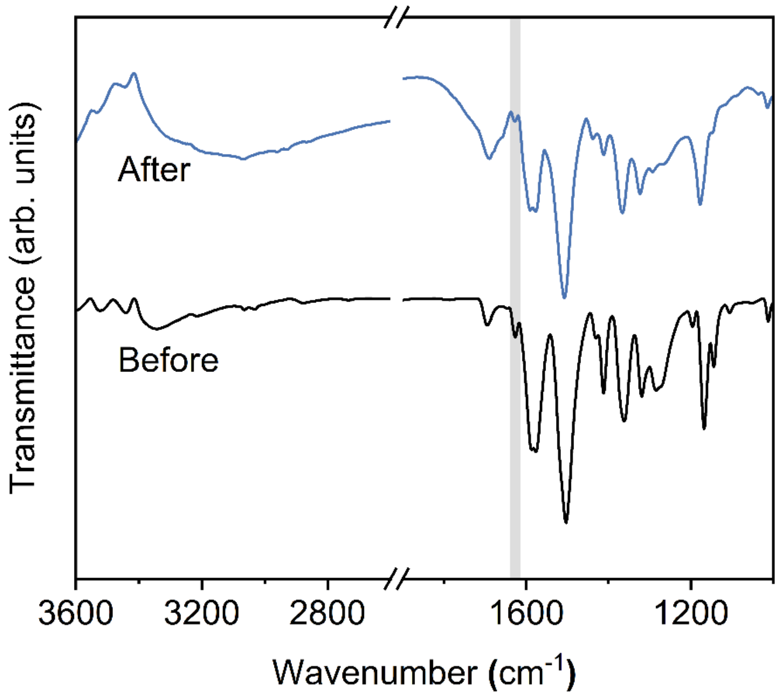

Supplementary Figure 38. FT-IR spectra of TFPA-TAPT-COF before and after five runs of sulfide oxidation.

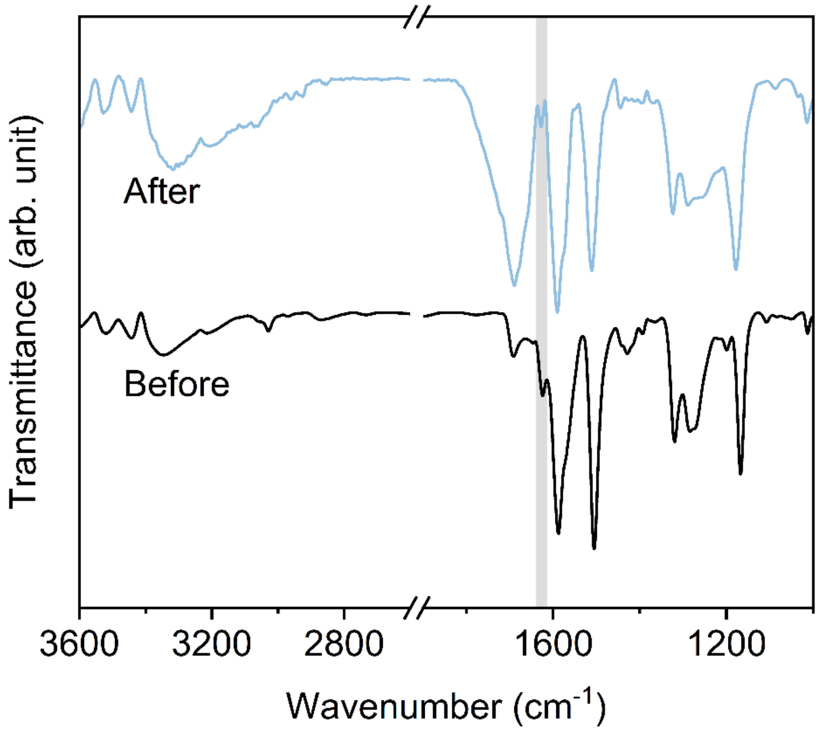

Supplementary Figure 39. FT-IR spectra of TFPA-TPB-COF before and after five runs of sulfide oxidation.

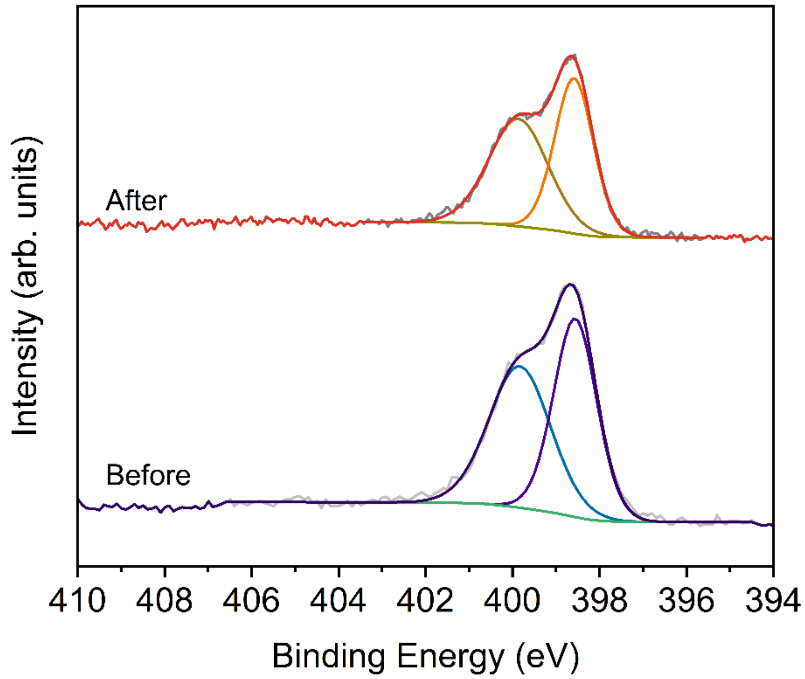

Supplementary Figure 40. XPS spectra of TFPA-TAPT-COF-Q before and after five runs of sulfide oxidation.

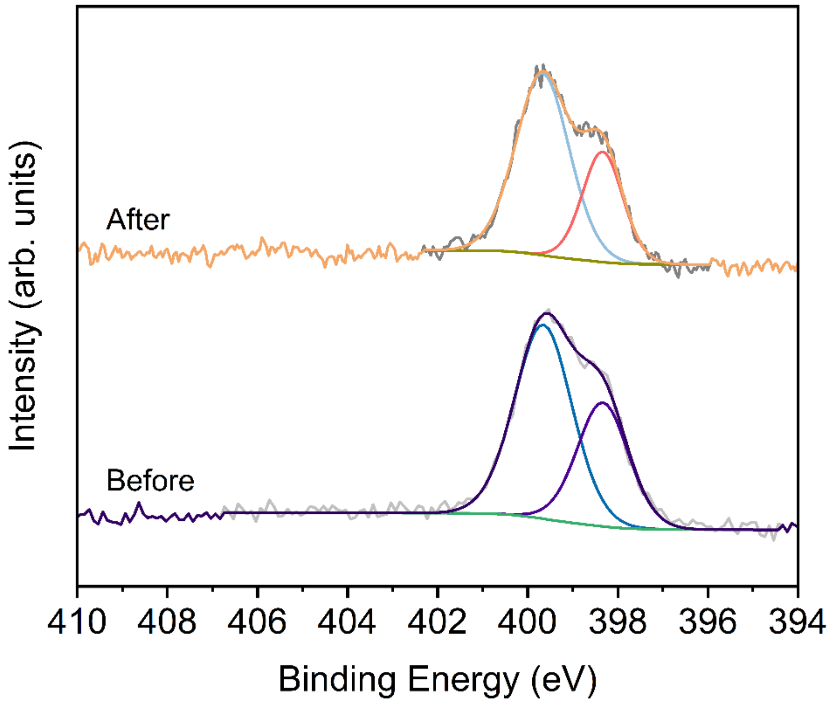

Supplementary Figure 41. XPS spectra of TFPA-TPB-COF-Q before and after five runs of sulfide oxidation.

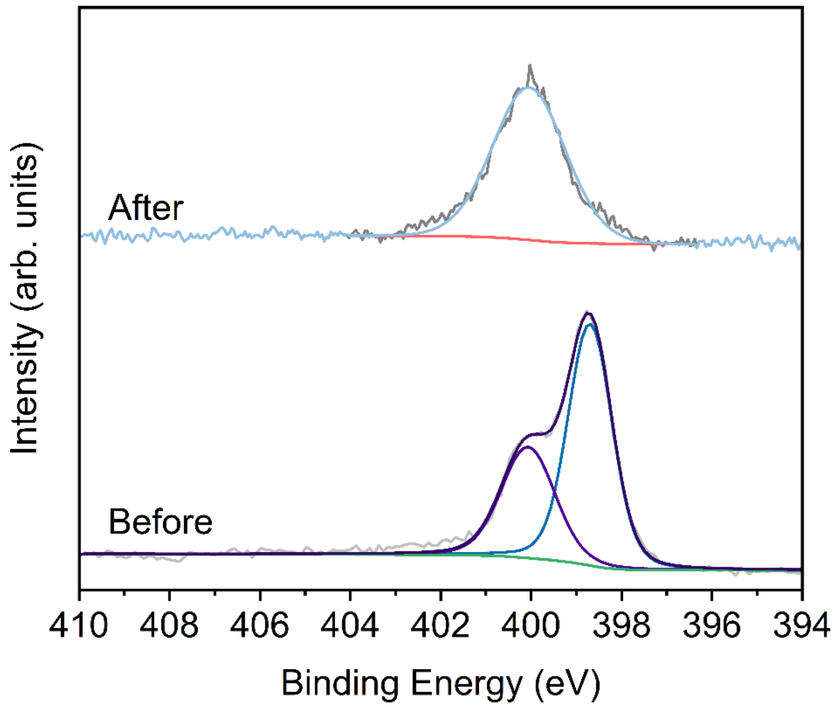

Supplementary Figure 42. XPS spectra of TFPA-TAPT-COF before and after five runs of sulfide oxidation.

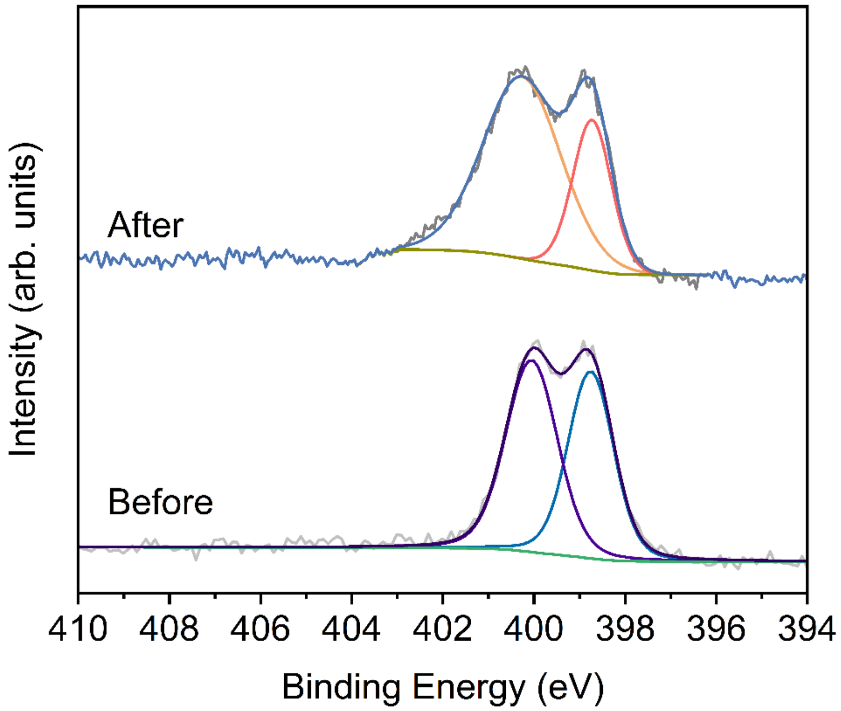

Supplementary Figure 43. XPS spectra of TFPA-TPB-COF before and after five runs of sulfide oxidation.

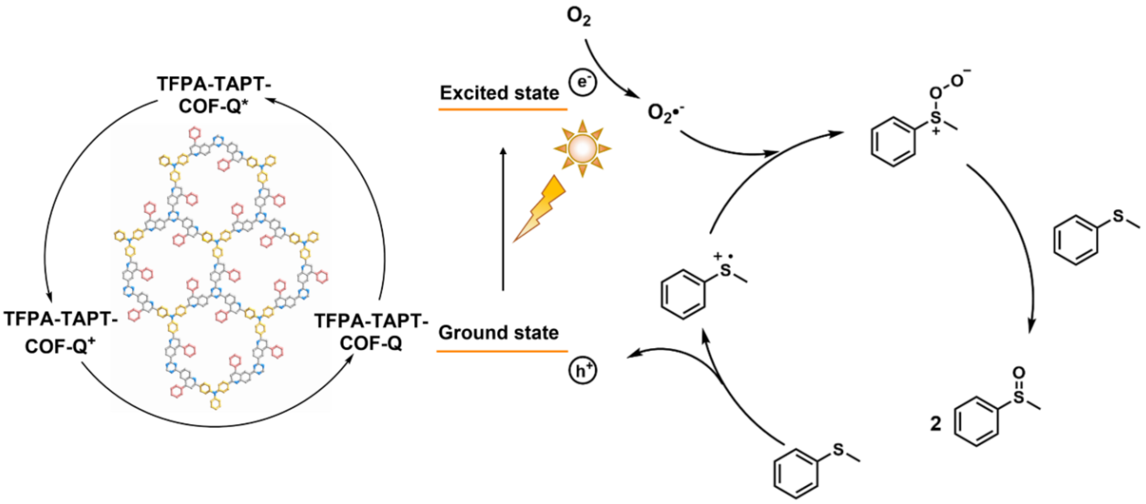

Supplementary Figure 44. Proposed mechanism of sulfide oxidation. the ground state of TFPA-TAPT-COF-Q is initially converted into excited state TFPA-TAPT-COF-Q* when it was excited by the irradiation light. Then, the excited electron is transferred to the oxygen molecule to give the generation of $O_2{\bullet}^-$ and a hole on TFPA-TAPT-COF-Q^+ is generated after TFPA-TAPT-COF-Q*, which could oxidize the substrate into the radical cation with itself being reduced to its original ground state (TFPA-TAPT-COF-Q). Subsequently, the nucleophilic addition happens on resulting radical cations by the generated $O_2{\bullet}^-$, converting to the products finally.

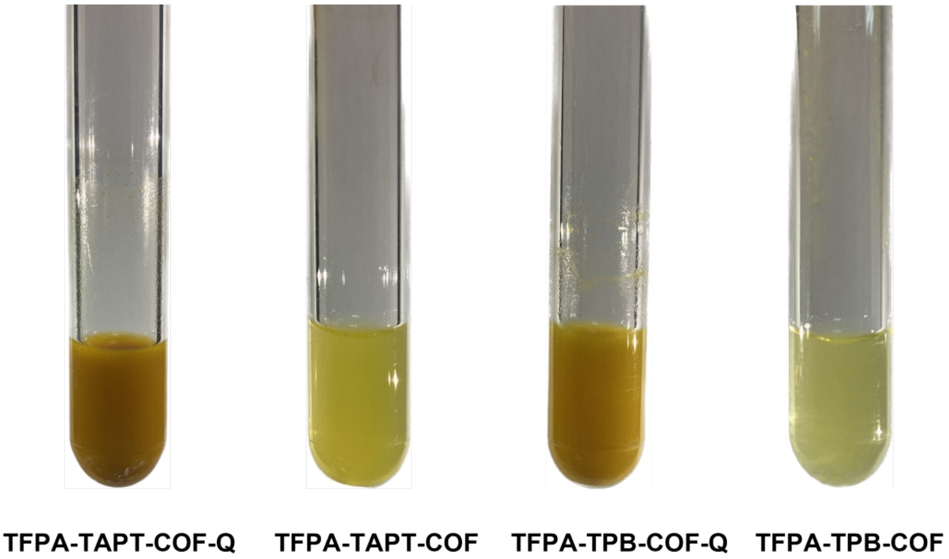

Supplementary Figure 45. Photos of TFPA-TAPT-COF-Q, TFPA-TAPT-COF, TFPA-TAPT-COF-Q and TFPA-TAPT-COF after the first run of oxidative decarboxylation of arylacetic acids.

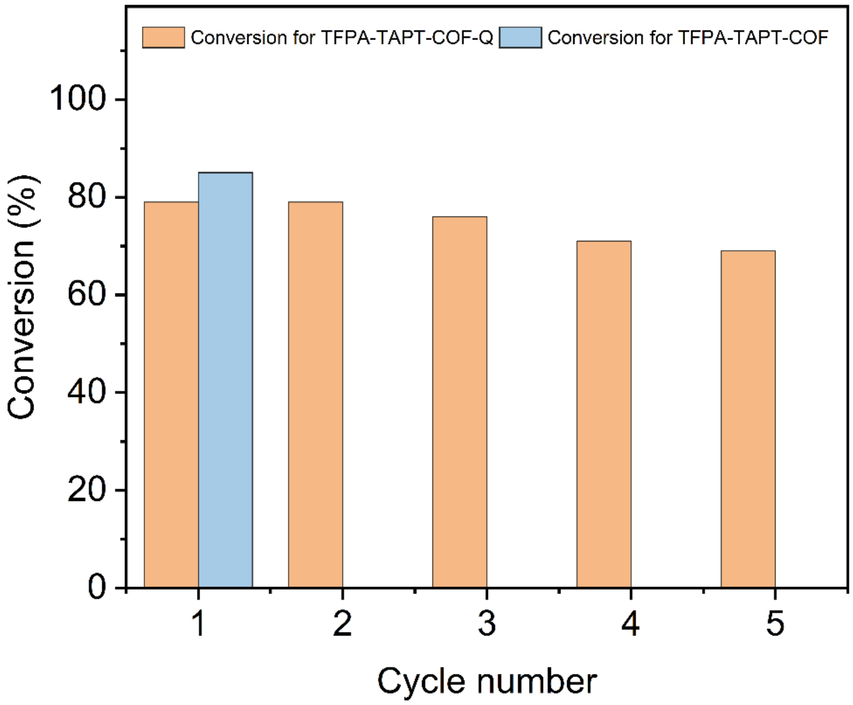

Supplementary Figure 46. Conversions over five runs of decarboxylation of arylacetic acids with TFPA-TPB-COF-Q (orange) and TFPA-TPB-COF (blue) as photocatalysts.

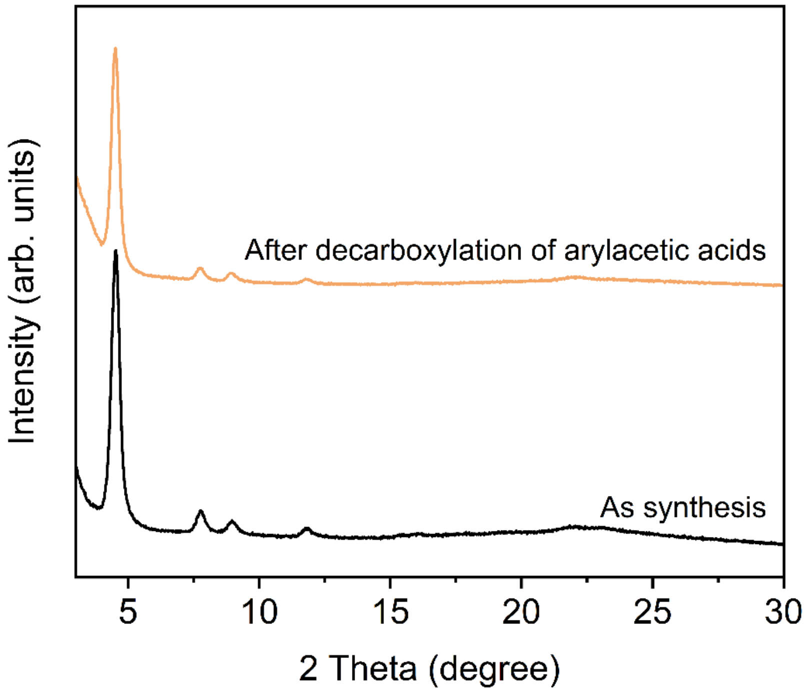

Supplementary Figure 47. PXRD patterns of TFPA-TPB-COF-Q before and after five runs of decarboxylation of arylacetic acids.

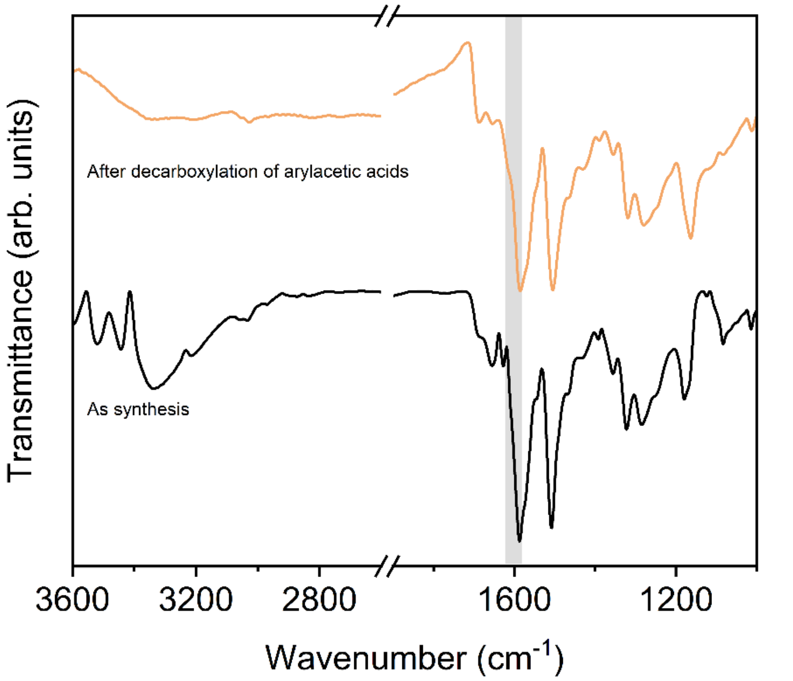

Supplementary Figure 48. FT-IR spectra of TFPA-TPB-COF-Q before and after five runs of decarboxylation of arylacetic acids.

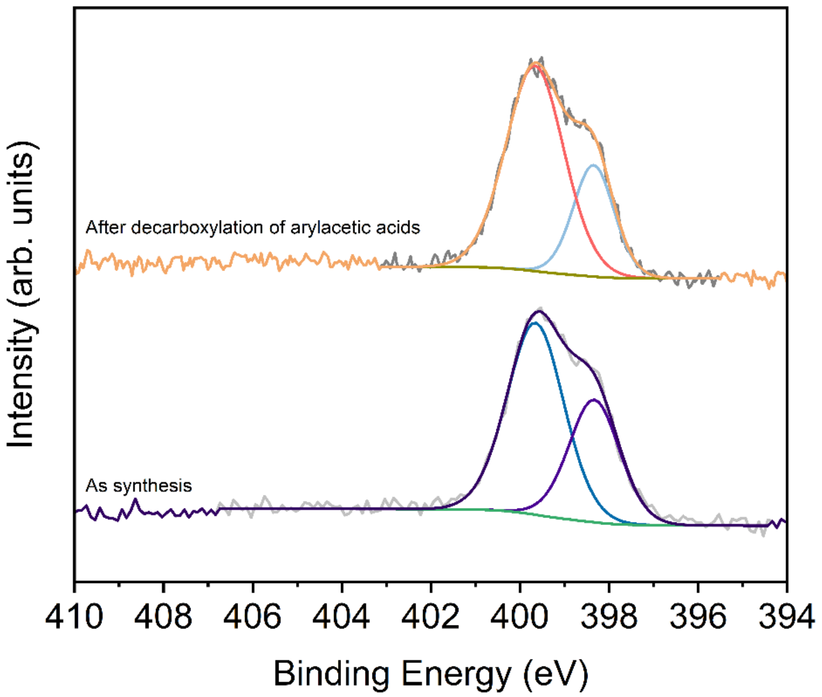

Supplementary Figure 49. XPS spectra of TFPA-TPB-COF-Q before and after five runs of decarboxylation of arylacetic acids.

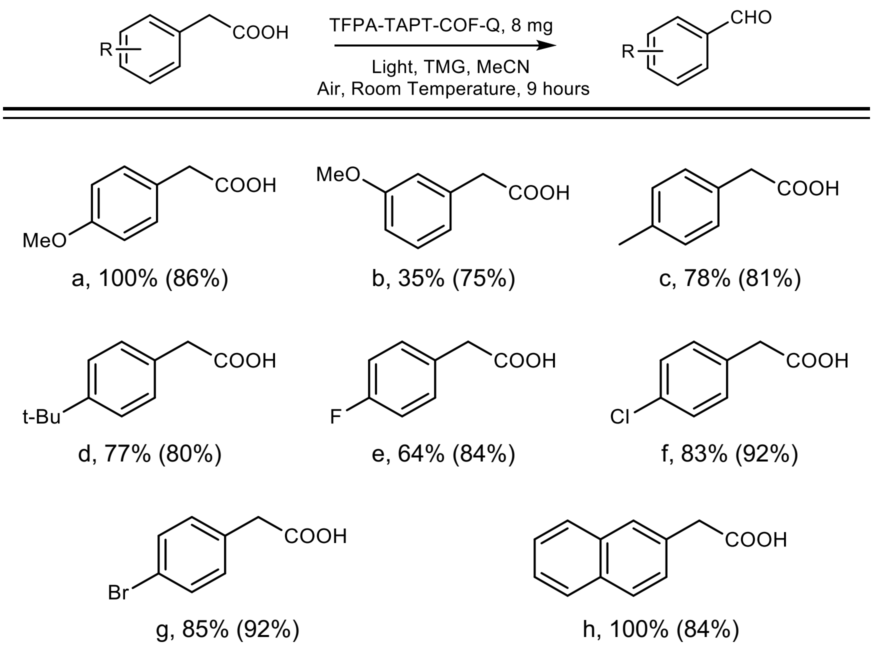

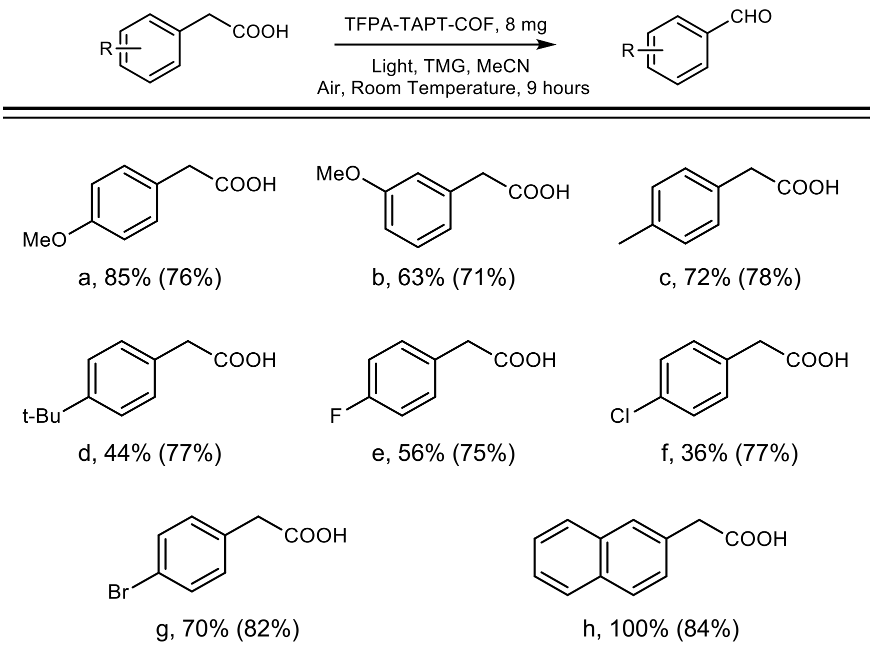

Supplementary Figure 50. **Photocatalytic results.** Photocatalytic results for different substrates of TFPA-TAPT-COF-Q (up) and TFPA-TAPT-COF (down) in oxidative decarboxylation of arylacetic acids (TMG means 1,1,3,3-tetramethylguanidine).

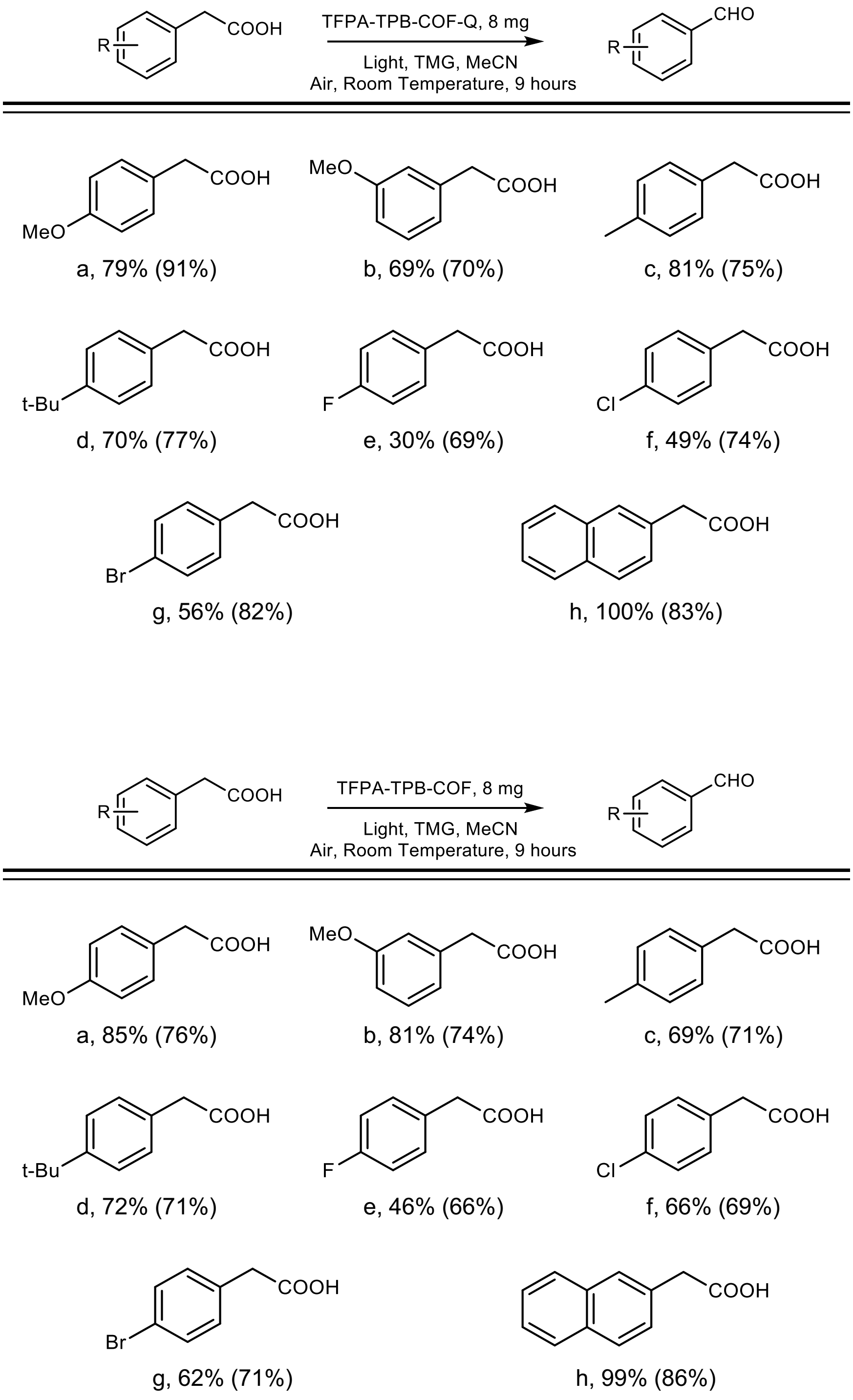

Supplementary Figure 51. **Photocatalytic results.** Photocatalytic results for different substrates of TFPA-TPB-COF-Q (up) and TFPA-TPB-COF (down) in oxidative decarboxylation of arylacetic acids (TMG means 1,1,3,3-tetramethylguanidine).

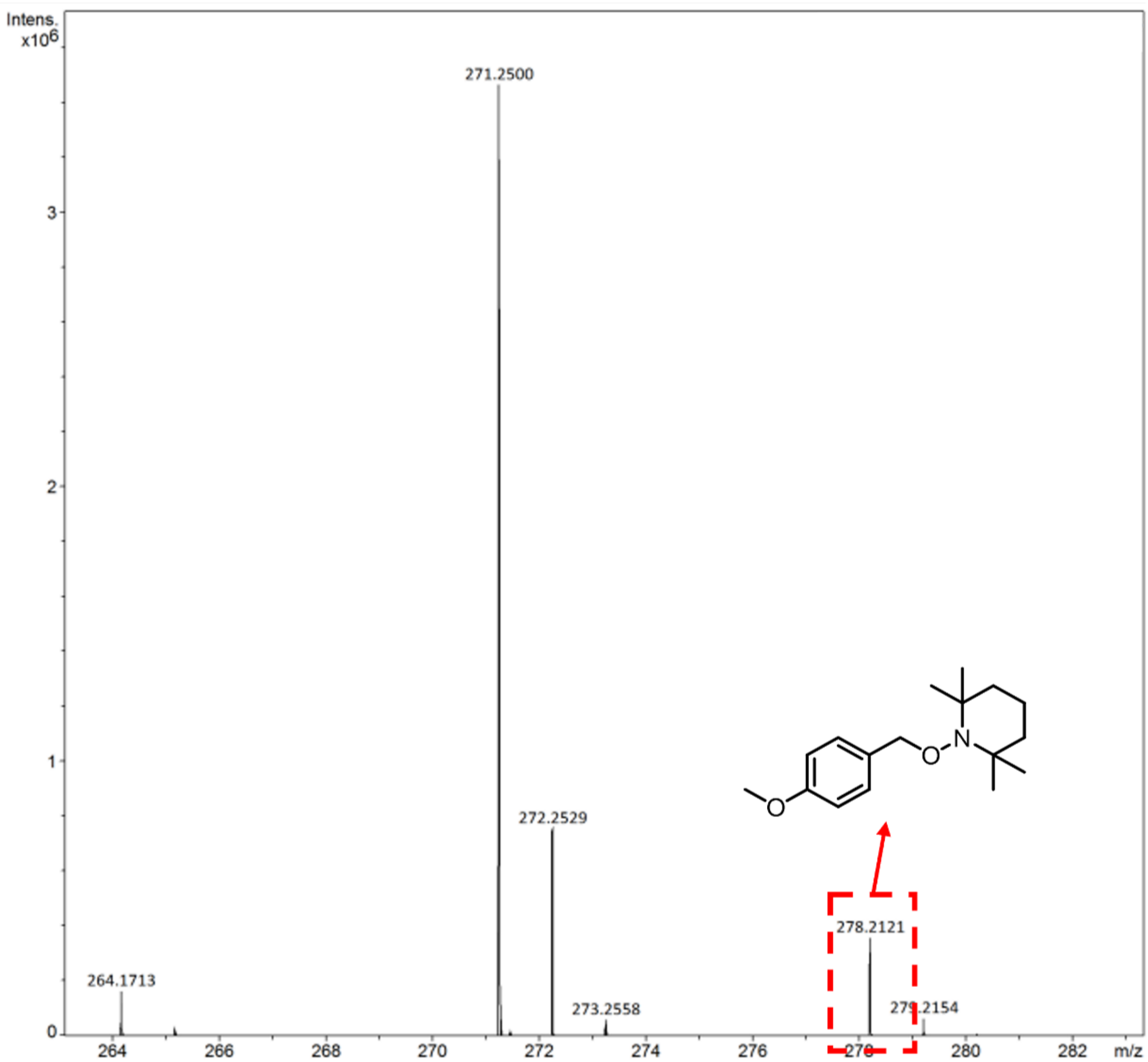

Supplementary Figure 52. HRMS of TEMPO trap experiment (crude reaction mixture was submitted for analysis). For TEMPO trapping experiments, 4-methoxyphenylacetic acid (33.2 mg, 0.2 mmol, 1.0 equiv), 1,1,3,3-tetramethyl guanidine (TMG) (50 μL, 0.4 mmol, 200 mol%) and (2,2,6,6-tetramethylpiperidin-1-yl)oxyl (TEMPO) (37.5 mg, 0.24 mmol, 1.2 equiv) were dissolved in dry MeCN (2.0 mL). The mixture was irradiated at 25W blue LED under air atmosphere for 9 h at room temperature (30 °C).[20]

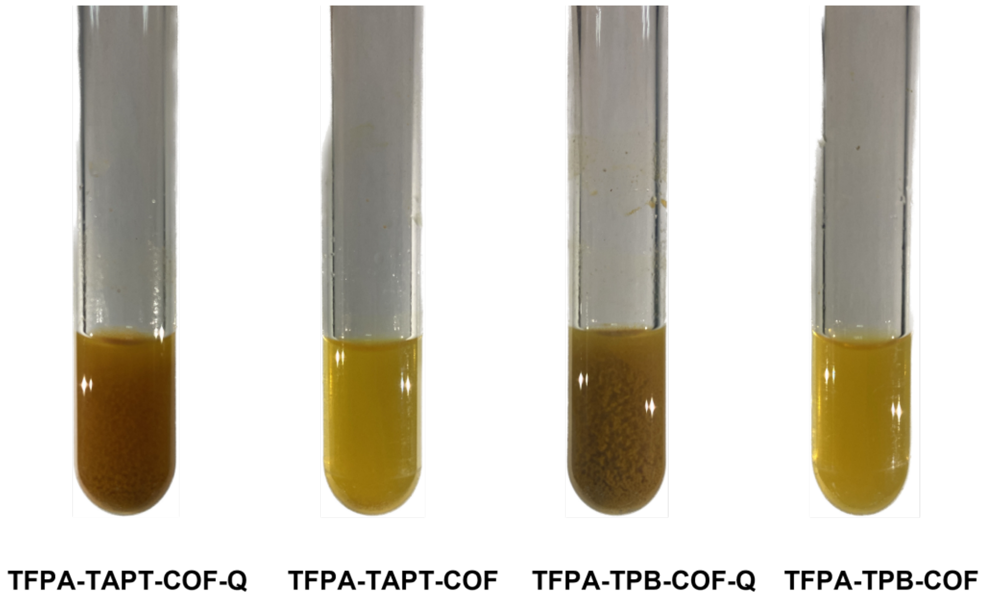

Supplementary Figure 53. Photos of TFPA-TAPT-COF-Q, TFPA-TAPT-COF, TFPA-TAPT-COF-Q and TFPA-TAPT-COF after the third run of benzylamine coupling.

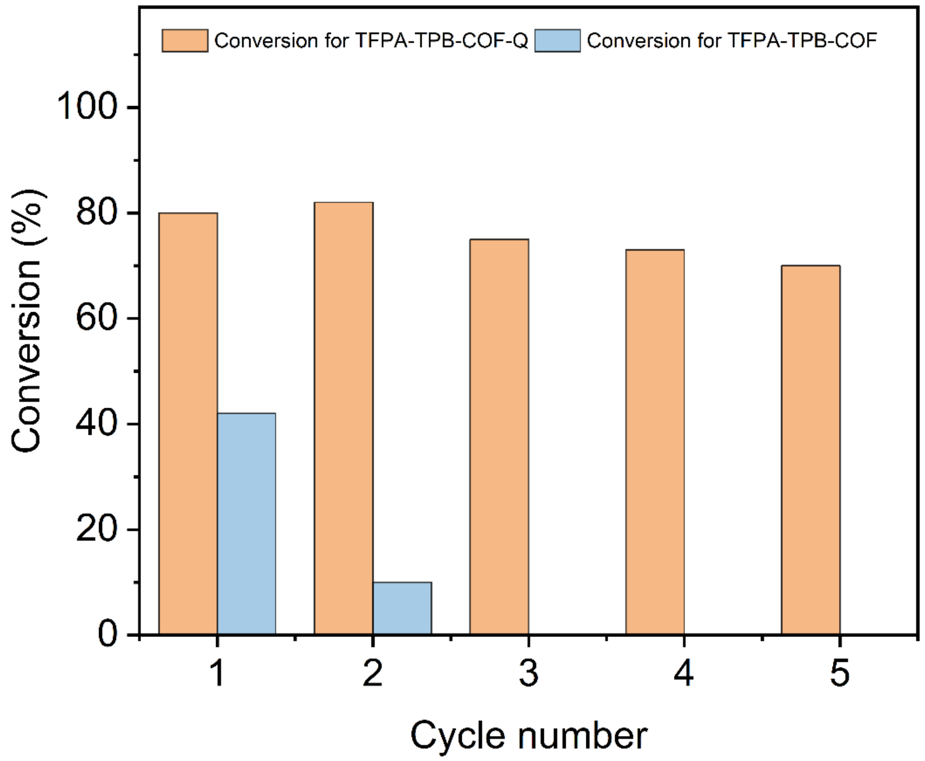

Supplementary Figure 54. Conversion over five runs of benzylamine coupling with TFPA-TPB-COF-Q (orange) and TFPA-TPB-COF (blue) as photocatalysts.

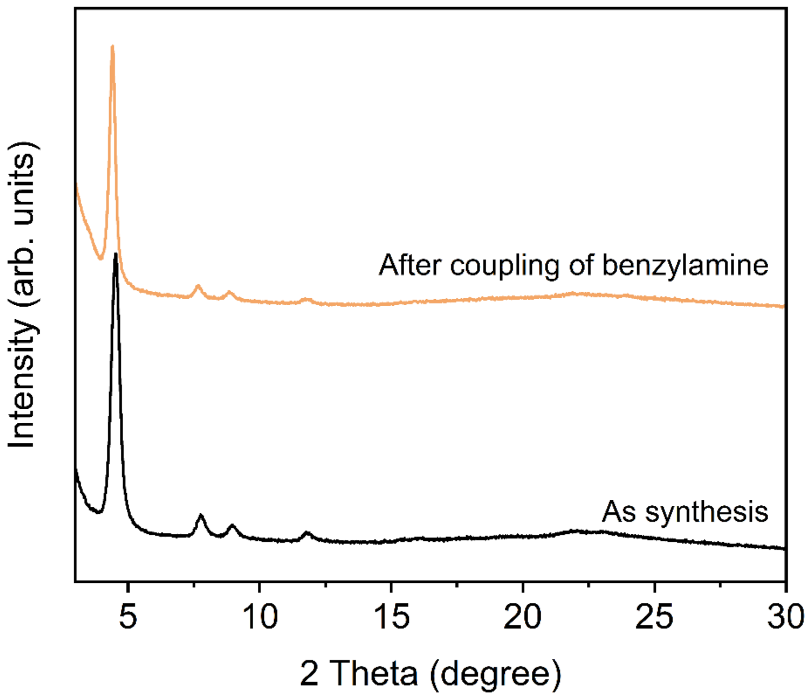

Supplementary Figure 55. PXRD patterns of TFPA-TPB-COF-Q before and after five runs of benzylamine coupling.

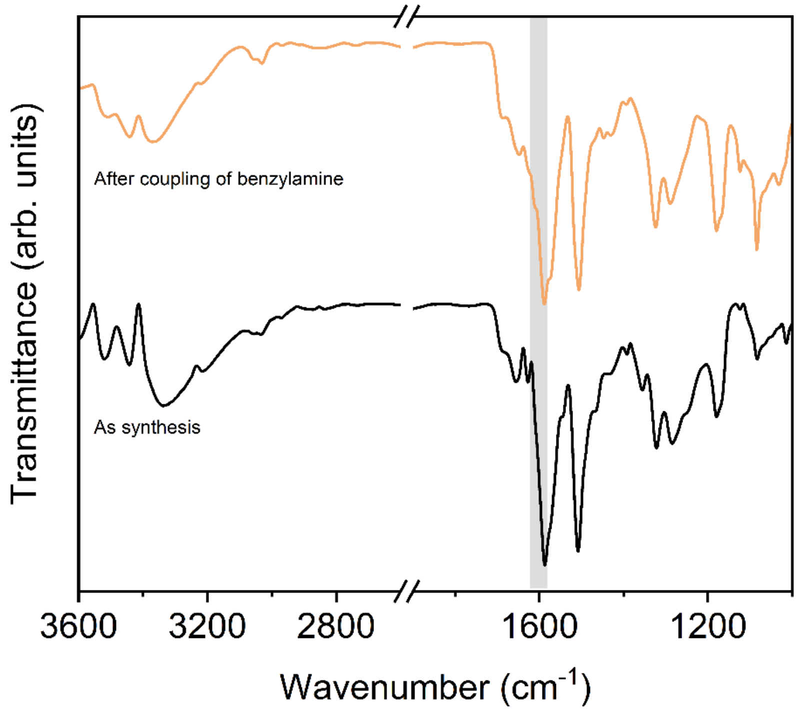

Supplementary Figure 56. FT-IR spectra of TFPA-TPB-COF-Q before and after five runs of benzylamine coupling.

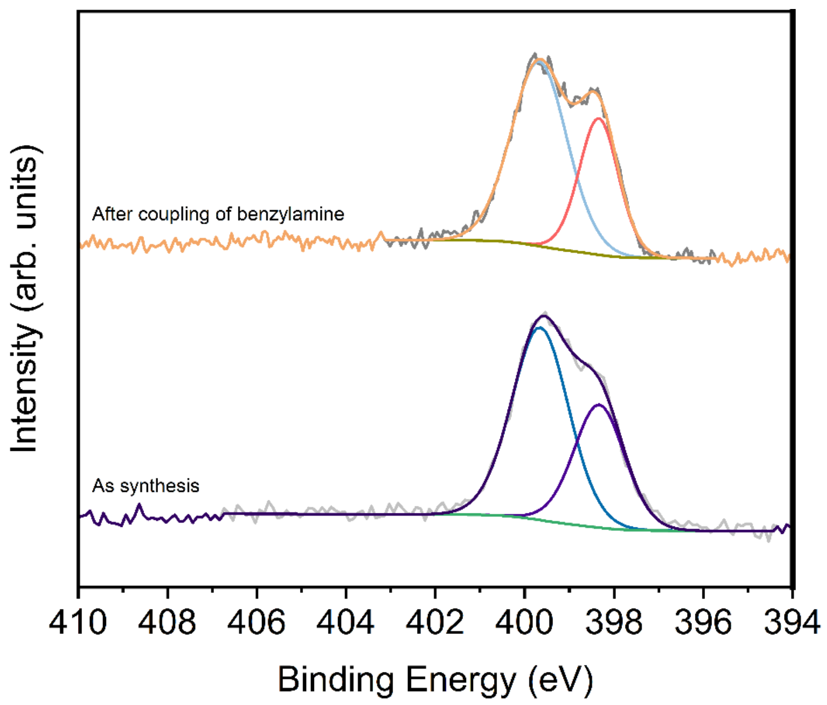

Supplementary Figure 57. XPS spectra of TFPA-TPB-COF-Q before and after five runs of benzylamine coupling.

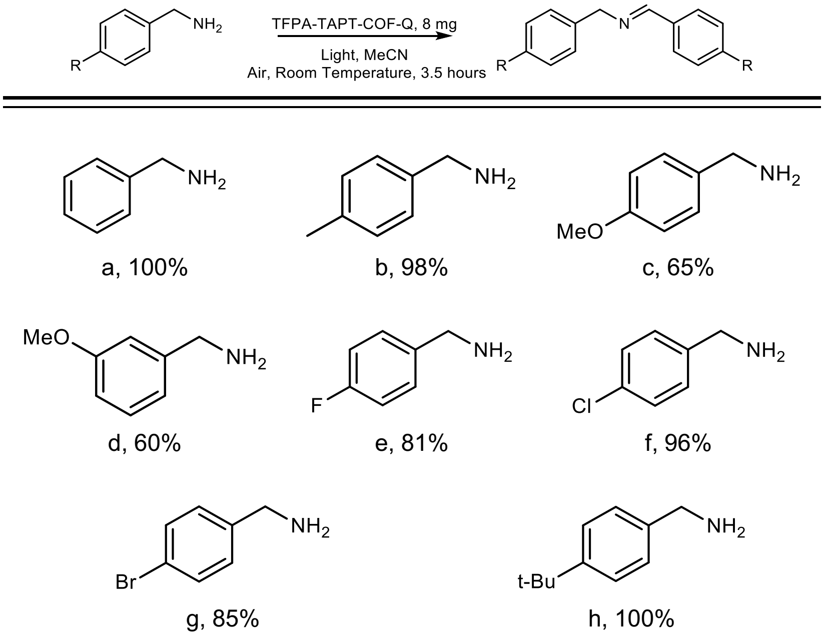

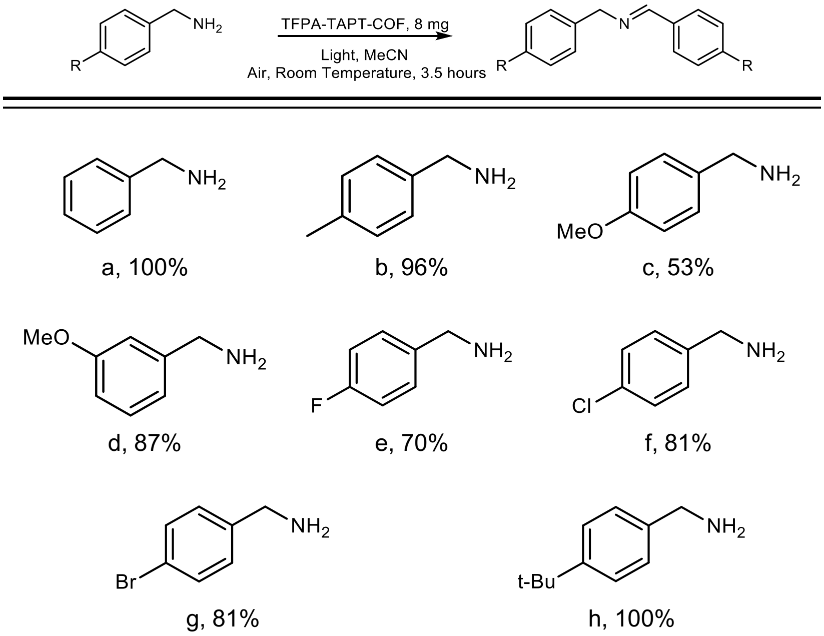

Supplementary Figure 58. **Photocatalytic results.** Photocatalytic results for different substrates of TFPA-TAPT-COF-Q (up) and TFPA-TAPT-COF (down) in coupling of benzylamines.

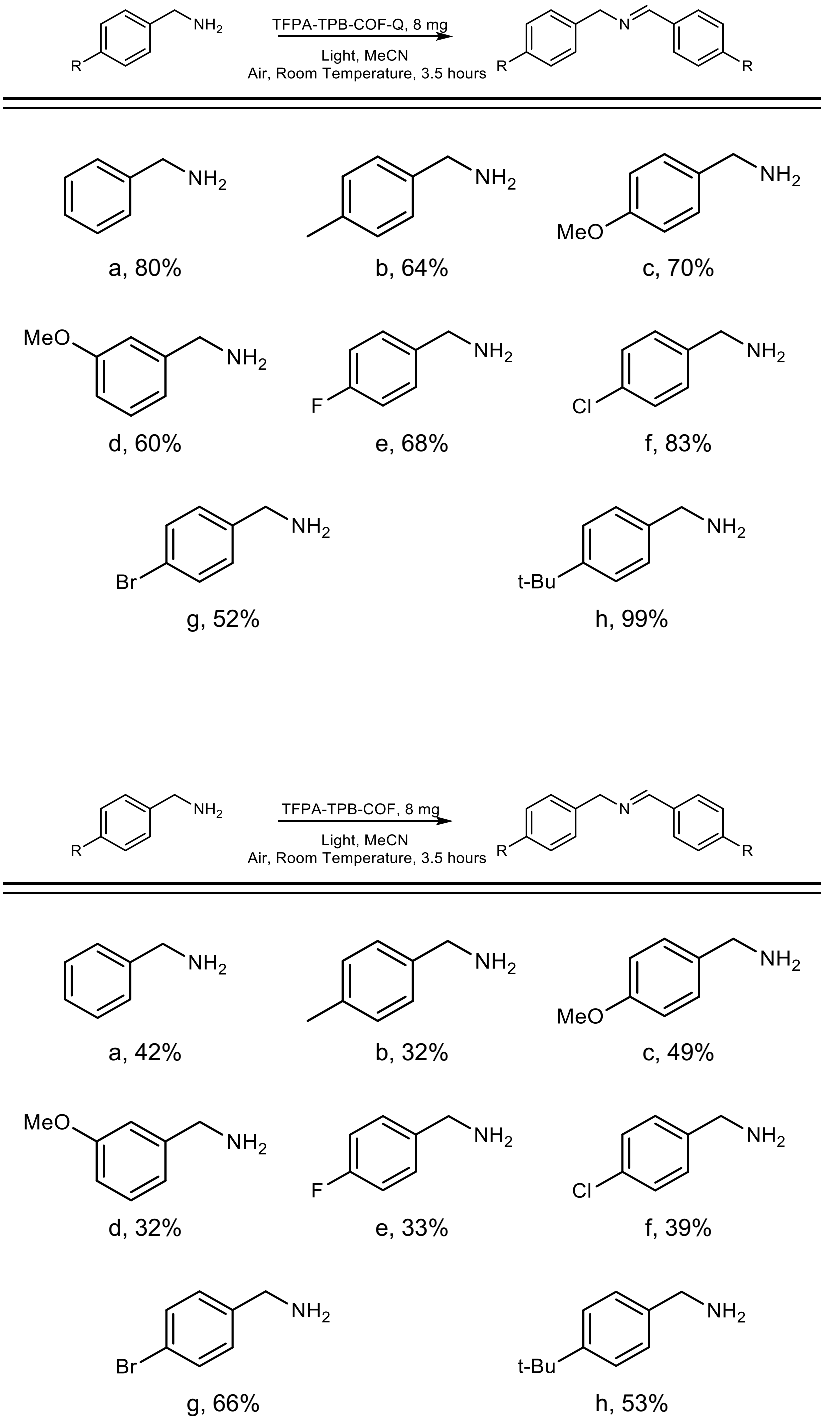

Supplementary Figure 59. **Photocatalytic results.** Photocatalytic results for different substrates of TFPA-TPB-COF-Q (up) and TFPA-TPB-COF (down) in coupling of benzylamines.

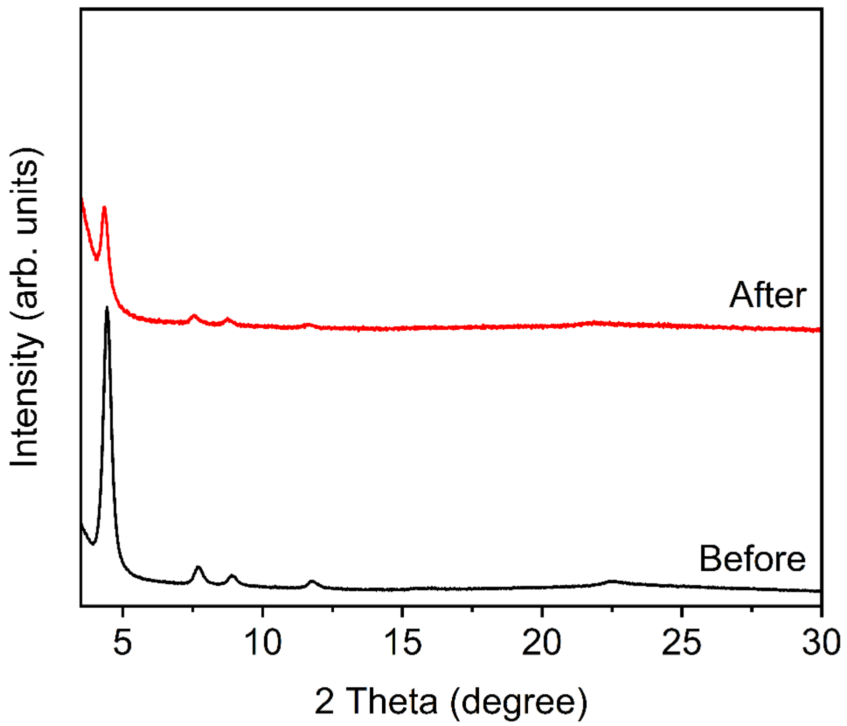

Supplementary Figure 60. PXRD patterns of TFPA-TAPT-COF-Q before and after 72 h photocatalytic H_2O_2 production.

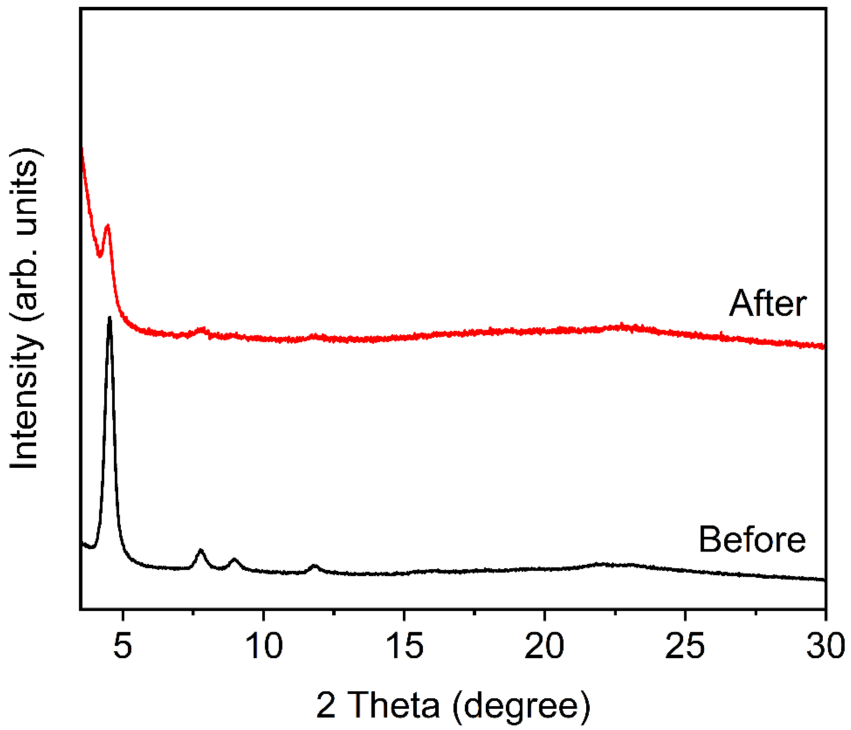

Supplementary Figure 61. PXRD patterns of TFPA-TPB-COF-Q before and after 72 h photocatalytic H_2O_2 production.

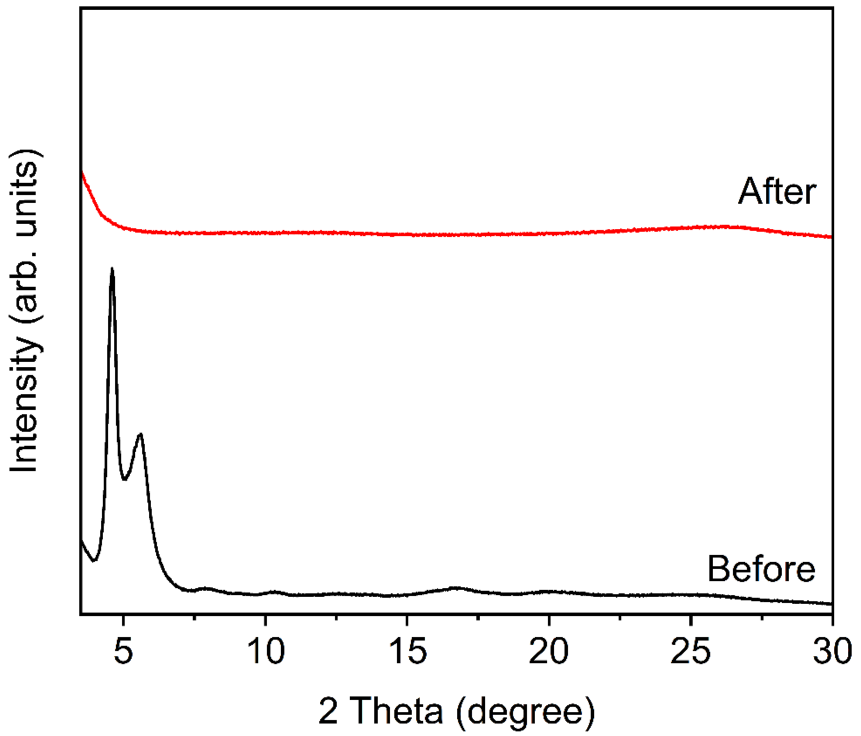

Supplementary Figure 62. PXRD patterns of TFPA-TAPT-COF before and after 24 h photocatalytic H_2O_2 production.

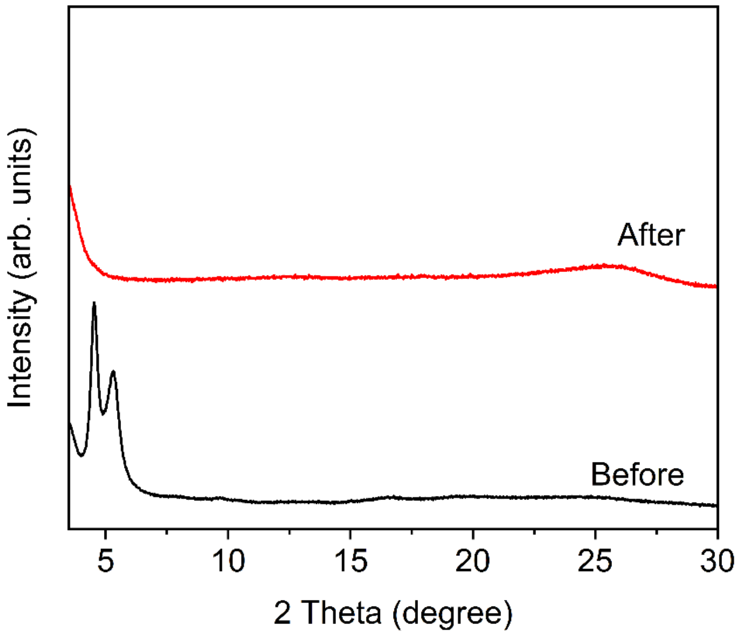

Supplementary Figure 63. PXRD patterns of TFPA-TPB-COF before and after 24 h photocatalytic H_2O_2 production.

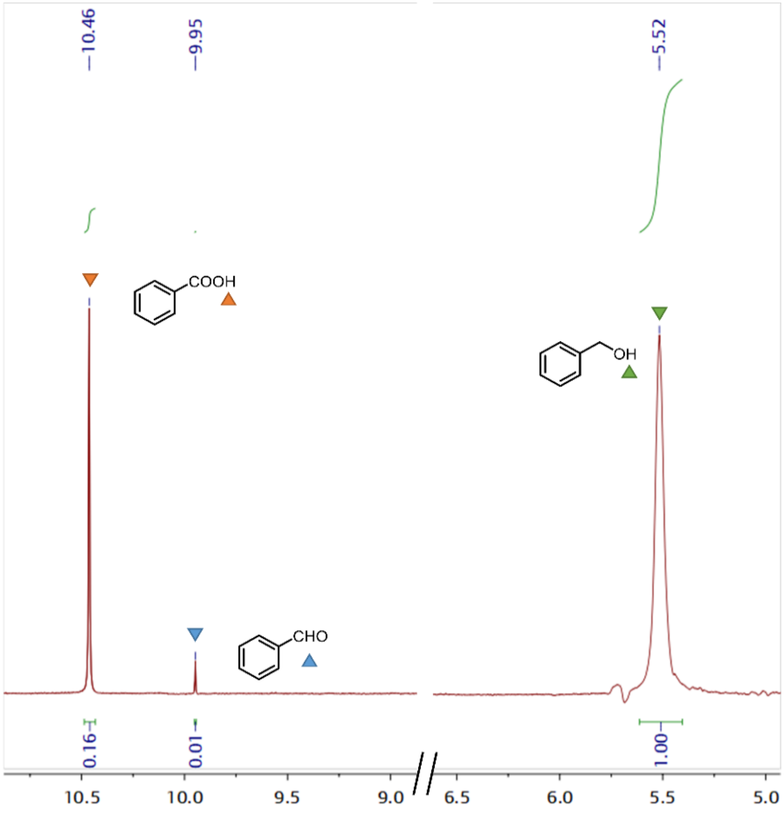

Supplementary Figure 64. ^{1}H NMR spectrum after photocatalytic H_2O_2 production showing the formation of benzaldehyde and benzoic acid from BA.

Supplementary Section 2: Characterization of Photocatalytic Products

Sulfide Oxidation:

(Methylsulfinyl)benzene

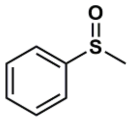

The title compound was made by following the general procedure using thioanisole (23 μL, 0.20 mmol) as the starting material. The product was obtained as a colorless liquid. ^{1}H NMR (400 MHz, $CDCl_3$): δ 7.68 (d, J = 2.5 Hz, 2H), 7.59-7.51 (m, 3H), 2.75 (s, 3H).

1-Fluoro-4-(methylsulfinyl)benzene

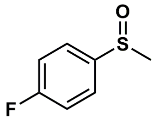

The titled compound was made by following the general procedure using 4-fluorothioanisole (24 μL, 0.20 mmol) as starting material. The product was obtained as a colorless liquid. ^{1}H NMR (400 MHz, $CDCl_3$): δ 7.59 (d, J = 4.4 Hz, 2H), 7.17 (d, J = 2.7 Hz, 2H), 2.68 (s, 3H).

1-Chloro-4-(methylsulfinyl)benzene

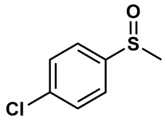

The titled compound was made by following the general procedure using (4-chlorophenyl)-(methyl)sulfane (26μL, 0.20 mmol) as starting material. The product was obtained as a colorless liquid. ^{1}H NMR (400 MHz, $CDCl_3$): δ 7.52 (d, J = 8.4 Hz, 2H), 7.44 (d, J = 8.6 Hz, 2H), 2.64 (s, 3H).

1-Bromo-4-methylsulfinylbenzene

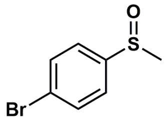

The titled compound was made by following the general procedure using 4-bromothioanisole (41 mg, 0.20 mmol) as starting material. The product was obtained as a colorless liquid. ^{1}H NMR (400 MHz, $CDCl_3$): δ 7.59 (d, J = 8.2 Hz, 2H), 7.45 (d, J = 8.4 Hz, 2H), 2.63 (s, 3H).

1-Methyl-4-(methylsulfinyl)benzene

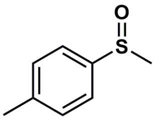

The title compound was made by following the general procedure using methyl(p-tolyl)sulfane (26 μL, 0.20 mmol) as starting material. The product was obtained as a colorless liquid.^{1}H NMR (400 MHz, $CDCl_3$): δ 7.45 (d, J = 8.2 Hz, 2H), 7.26 (d, J = 7.9 Hz, 2H), 2.62 (s, 3H), 2.32 (s, 3H).

1-Methanesulfinyl-4-methoxybenzene

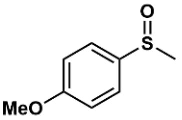

The title compound was made by following the general procedure using *p*-methylthioanisole (46 mg, 0.25 mmol) as starting material. The product was obtained as a colorless liquid. ^{1}H NMR (400 MHz, $CDCl_3$) δ 7.51 (d, J = 8.4 Hz, 2H), 6.96 (d, J = 5.5 Hz, 2H), 3.79 (s, 3H), 2.65 (s, 3H).

Ethylsulfinylbenzene

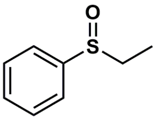

The title compound was made by following the general procedure using phenylethylthiol (27 μL, 0.20 mmol) as starting material. The product was obtained as a colorless liquid. ^{1}H NMR (400 MHz, $CDCl_3$): δ 7.49 (d, J = 7.9 Hz, 2H), 7.41 (d, J = 7.2 Hz, 3H), 3.01 (d, J = 7.4 Hz, 2H), 1.05 (s, 3H).

1-(Benzenesulfinyl)-4-methylbenzene

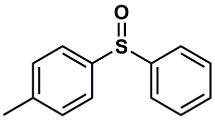

The title compound was made by following the general procedure using 4-methylphenyl phenyl sulfide (37 μL, 0.20 mmol) as starting material. The product was obtained as a colorless liquid. ^{1}H NMR (400 MHz, $CDCl_3$): δ 7.47 (d, J = 7.4 Hz, 2H), 7.40 – 7.29 (m, 5H), 7.13 (d, J = 7.8 Hz, 2H), 2.20 (s, 3H).

1-Chloro-2-ethylsulfinylethane

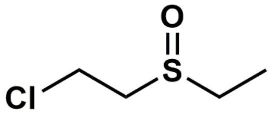

The title compound was made by following the general procedure using 2-chloroethyl ethyl sulfide (23 μL, 0.20 mmol) as starting material. The product was obtained as a colorless liquid. ^{1}H NMR (400 MHz, $CDCl_3$): δ 3.81 (dd, J = 6.3, 3.0 Hz, 2H), 2.91 (t, J = 7.4 Hz, 2H), 2.70 (d, J = 3.6 Hz, 2H), 1.46 (t, J = 7.3 Hz, 3H).

Oxidative decarboxylation of arylacetic acids

4-methoxybenzaldehyde

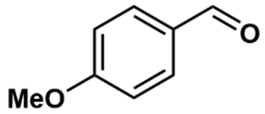

The title compound was made by following the general procedure using 4-methoxyphenylaceticacid (33.2 mg, 0.2 mmol) as starting material. ^{1}H NMR (400 MHz, Chloroform-d) δ 9.87 (s, 1H), 7.83 (d, J = 8.8 Hz, 2H), 6.99 (d, J = 8.8 Hz, 2H), 3.87 (s, 3H).

4-methylbenzaldehyde

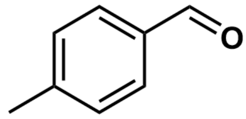

The title compound was made by following the general procedure using 4-methylphenylaceticacid (30.0 mg, 0.2 mmol) as starting material. ^{1}H NMR (400 MHz, Chloroform-d) δ 9.94 (s, 1H), 7.76 (d, J = 8.2 Hz, 2H), 7.31 (d, J = 7.8 Hz, 2H), 2.41 (s, 3H).

4-(t-Butyl)benzaldehyde

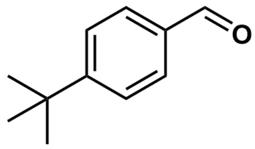

The title compound was made by following the general procedure using 4-(t-Butyl)phenylaceticacid (38.4 mg, 0.2 mmol) as starting material. ^{1}H NMR (400 MHz, Chloroform-d) δ 9.98 (s, 1H), 7.82 (d, J = 8.3 Hz, 2H), 7.55 (d, J = 8.4 Hz, 2H), 1.35 (s, 9H).

4-fluorobenzaldehyde

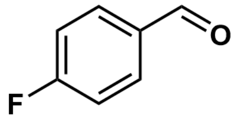

The title compound was made by following the general procedure using 4-fluorophenylaceticacid (30.8 mg, 0.2 mmol) as starting material. ^{1}H NMR (400 MHz, Chloroform-d) δ 9.95 (s, 1H), 7.88 (m, 2H), 7.20 (d, J = 8.0 Hz, 7.5 Hz, 2H).

4-chlorobenzaldehyde

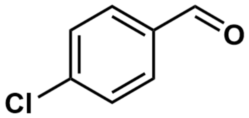

The title compound was made by following the general procedure using 4-chlorophenylaceticacid (34.1 mg, 0.2 mmol) as starting material. ^{1}H NMR (400 MHz, Chloroform-d) δ 9.97 (s, 1H), 7.83 (d, J = 8.6 Hz, 2H), 7.50 (d, J = 8.4 Hz, 2H).

4-bromobenzaldehyde

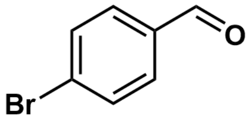

The title compound was made by following the general procedure using 4-bromophenylaceticacid (43.0 mg, 0.2 mmol) as starting material. ^{1}H NMR (400MHz, CDCl3) δ 10.00 (s, 1H), 7.77 (d, H=8.0 Hz, 2H), 7.71 (d, J=8.0 Hz, 2H).

2-naphthaldehyde

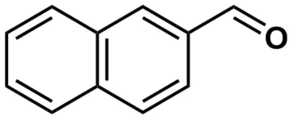

The title compound was made by following the general procedure using 2-Naphthaleneacetic acid (37.2 mg, 0.2 mmol) as starting material. ^{1}H NMR (400 MHz, Chloroform-d) δ 10.15 (s, 1H), 8.33 (s, 1H), 8.12-7.84 (m, 4H), 7.61 (m, 2H).

Coupling of benzylamine

N-benzyl-1-phenylmethanimine

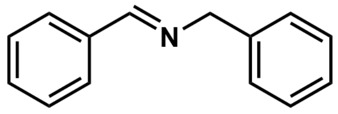

The title compound was made by following the general procedure using benzylamine (22 μL, 0.2 mmol) as starting material. ^{1}H NMR (400 MHz, $CDCl_3$) 4.81 (2H, s), 7.26-7.33 (8H, m), 7.68-7.87 (2H, m), 8.38 (1H, s).

N-(4-methylbenzyl)-1-(*p*-tolyl)methanimine

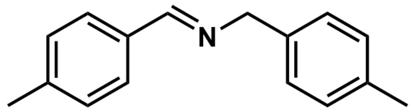

The title compound was made by following the general procedure using 4-methylbenzylamine (25 μL, 0.2 mmol) as starting material. ^{1}H NMR (400 MHz, $CDCl_3$) 2.24 (3H, s), 2.28 (3H, s), 4.67 (2H, s), 7.04-7.14 (6H, m), 7.57 (2H, d, J = 8.0 Hz), 8.24 (1H, s).

N-(4-butylbenzyl)-1-(4-butylphenyl)methanimine

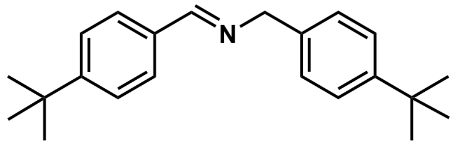

The title compound was made by following the general procedure using 4-butylbenzylamine (35 μL, 0.2 mmol) as starting material. ^{1}H NMR (400 MHz, $CDCl_3$) 0.97-1.01 (6H, m), 1.37-1.46 (4H, m), 1.61-1.71 (4H, m), 2.64-2.71 (4H, m), 4.83 (2H, s), 7.21 (2H, d, J = 8.0 Hz), 7.26-7.31 (4H, m), 7.75 (2H, d, J = 8.4 Hz), 8.40 (1H, s).

N-(4-methoxybenzyl)-1-(4-methoxyphenyl)methanimine

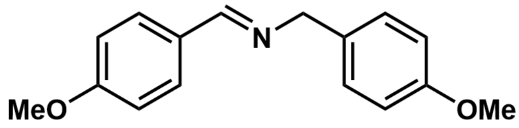

The title compound was made by following the general procedure using 4-methoxybenzylamine (26 μL, 0.2 mmol) as starting material. ^{1}H NMR (400 MHz, $CDCl_3$) 3.70 (3H, s), 3.74 (3H, s), 4.64 (2H, s), 6.79 (2H, d, J = 8.4 Hz), 6.84 (2H, d, J = 8.8 Hz), 7.26 (2H, d, J =8.8 Hz), 7.63 (2H, d, J = 8.8 Hz), 8.21 (1H, s).

N-(4-fluorobenzyl)-1-(4-fluorophenyl)methanimine

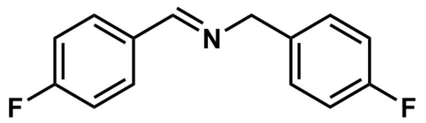

The title compound was made by following the general procedure using 4-fluorobenzylamine (23 μL, 0.2 mmol) as starting material. ^{1}H NMR (400 MHz, $CDCl_3$) 4.67 (2H, s), 6.94 (2H, dddd, J = 2.0, 2.8, 8.4, 8.8 Hz), 7.01 (2H, dddd, J = 2.0, 2.8, 8.4, 8.8 Hz), 7.20 (2H, dddd, J = 2.0, 2.8, 5.2, 8.4 Hz), 7.68 (2H, dddd, J = 2.0, 2.8, 5.2, 8.4 Hz), 8.25 (1H, s).

N-(4-chlorobenzyl)-1-(4-chlorophenyl)methanimine

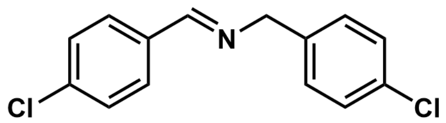

The title compound was made by following the general procedure using 4-chlorobenzylamine (24 μL, 0.2 mmol) as starting material. ^{1}H NMR (400 MHz, $CDCl_3$) 4.75 (2H, s), 7.21-7.31 (6H, m), 7.37 (2H, d, J = 8.8 Hz), 7.69 (2H, d, J = 8.4 Hz), 8.32 (1H, s).

N-(4-bromobenzyl)-1-(4-bromophenyl)methanimine

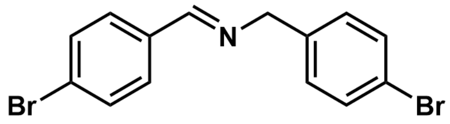

The title compound was made by following the general procedure using 4-bromobenzylamine (37.2 mg, 0.2 mmol) as starting material. ^{1}H NMR (400 MHz, $CDCl_3$) 4.87 (2H, s), 7.05 (6H, m), 7.65 (2H, d, J = 8.8 Hz), 7.77 (2H, d, J = 8.4 Hz), 8.54 (1H, s).

Supplementary Section 3: Structure Coordinates

Supplementary Table 8. Atomic Coordinates for Simulated Structure of TFPA-TAPT-COF-Q in AA Stacking Model.

C1	C	0.39014	-0.15349	-0.54830	0	Uiso	1
C2	C	0.35101	-0.22183	-0.55085	0	Uiso	1
C3	C	0.37444	-0.26201	-0.40994	0	Uiso	1
C4	C	0.43878	-0.23123	-0.26915	0	Uiso	1
C5	C	0.47843	-0.16293	-0.27155	0	Uiso	1
C6	C	0.45419	-0.12379	-0.40944	0	Uiso	1
C7	C	0.49657	-0.05223	-0.40654	0	Uiso	1
N8	N	0.51214	-0.47420	-0.49511	0	Uiso	1
C9	C	0.54218	-0.51422	-0.49959	0	Uiso	1
C10	C	0.50515	-0.58264	-0.45384	0	Uiso	1
C11	C	0.53592	-0.62035	-0.45992	0	Uiso	1
C12	C	0.60403	-0.59028	-0.51028	0	Uiso	1
C13	C	0.64084	-0.52199	-0.55616	0	Uiso	1
C14	C	0.61004	-0.48428	-0.55193	0	Uiso	1
C15	C	0.63657	-0.62997	-0.51306	0	Uiso	1
N16	N	0.60022	-0.69661	-0.51296	0	Uiso	1
C17	C	0.95827	0.56984	0.54616	0	Uiso	1
C18	C	1.03359	0.60143	0.54616	0	Uiso	1
C19	C	0.92535	0.61237	0.54616	0	Uiso	1
C20	C	0.84989	0.57927	0.54616	0	Uiso	1
C21	C	0.81391	0.61890	0.54616	0	Uiso	1
C22	C	0.85648	0.69434	0.54616	0	Uiso	1
C23	C	0.93175	0.72544	0.54616	0	Uiso	1
C24	C	0.96438	0.68266	0.54616	0	Uiso	1
H25	H	0.37004	-0.12069	-0.66091	0	Uiso	1
H26	H	0.29819	-0.24684	-0.66981	0	Uiso	1
H27	H	0.45872	-0.26361	-0.14981	0	Uiso	1
H28	H	0.53181	-0.13804	-0.15923	0	Uiso	1
H29	H	0.50469	-0.67777	-0.42264	0	Uiso	1
H30	H	0.69810	-0.49616	-0.59856	0	Uiso	1
H31	H	0.64120	-0.42695	-0.59256	0	Uiso	1
H32	H	1.06706	0.65874	0.51230	0	Uiso	1
H33	H	0.81743	0.52144	0.54616	0	Uiso	1

H34	H	0.75595	0.59132	0.54616	0	Uiso	1
H35	H	0.83129	0.72697	0.54616	0	Uiso	1
H36	H	0.96560	0.78313	0.54616	0	Uiso	1
H37	H	1.02219	0.70767	0.54616	0	Uiso	1
N38	N	0.33333	-0.33333	-0.40992	0	Uiso	1

Supplementary Table 9. Atomic Coordinates for Simulated Structure of TFPA-TAPT-COF-Q in AB Stacking Model.

C1	C	0.39014	0.84651	0.22585	0	Uiso	1
C2	C	0.35101	0.77817	0.22458	0	Uiso	1
C3	C	0.37444	0.73799	0.29503	0	Uiso	1
C4	C	0.43878	0.76877	0.36542	0	Uiso	1
C5	C	0.47843	0.83707	0.36423	0	Uiso	1
C6	C	0.45419	0.87621	0.29528	0	Uiso	1
C7	C	0.49657	0.94777	0.29673	0	Uiso	1
N8	N	0.51214	0.52580	0.25244	0	Uiso	1
C9	C	0.54218	0.48578	0.25020	0	Uiso	1
C10	C	0.50515	0.41736	0.27308	0	Uiso	1
C11	C	0.53592	0.37965	0.27004	0	Uiso	1
C12	C	0.60403	0.40972	0.24486	0	Uiso	1
C13	C	0.64084	0.47801	0.22192	0	Uiso	1
C14	C	0.61004	0.51572	0.22403	0	Uiso	1
C15	C	0.63657	0.37003	0.24347	0	Uiso	1
N16	N	0.60022	0.30339	0.24352	0	Uiso	1
C17	C	0.95827	0.56984	0.27308	0	Uiso	1
C18	C	0.03359	0.60143	0.27308	0	Uiso	1
C19	C	0.92535	0.61237	0.27308	0	Uiso	1
C20	C	0.84989	0.57927	0.27308	0	Uiso	1
C21	C	0.81391	0.61890	0.27308	0	Uiso	1
C22	C	0.85648	0.69434	0.27308	0	Uiso	1
C23	C	0.93175	0.72544	0.27308	0	Uiso	1
C24	C	0.96438	0.68266	0.27308	0	Uiso	1
H25	H	0.37004	0.87931	0.16954	0	Uiso	1
H26	H	0.29819	0.75316	0.16510	0	Uiso	1
H27	H	0.45872	0.73639	0.42510	0	Uiso	1
H28	H	0.53181	0.86196	0.42039	0	Uiso	1
H29	H	0.50469	0.32223	0.28868	0	Uiso	1
H30	H	0.69810	0.50384	0.20072	0	Uiso	1
H31	H	0.64120	0.57305	0.20372	0	Uiso	1
H32	H	0.06706	0.65874	0.25615	0	Uiso	1
H33	H	0.81743	0.52144	0.27308	0	Uiso	1
H34	H	0.75595	0.59132	0.27308	0	Uiso	1
H35	H	0.83129	0.72697	0.27308	0	Uiso	1
H36	H	0.96560	0.78313	0.27308	0	Uiso	1

H37	H	0.02219	0.70767	0.27308	0	Uiso	1
C38	C	0.05681	1.17985	0.72585	0	Uiso	1
C39	C	0.01767	1.11150	0.72458	0	Uiso	1
C40	C	0.04111	1.07132	0.79503	0	Uiso	1
C41	C	0.10545	1.10210	0.86542	0	Uiso	1
C42	C	0.14510	1.17041	0.86423	0	Uiso	1
C43	C	0.12086	1.20955	0.79528	0	Uiso	1
C44	C	0.16324	1.28110	0.79673	0	Uiso	1
N45	N	0.17881	0.85913	0.75244	0	Uiso	1
C46	C	0.20885	0.81911	0.75020	0	Uiso	1
C47	C	0.17182	0.75069	0.77308	0	Uiso	1
C48	C	0.20259	0.71298	0.77004	0	Uiso	1
C49	C	0.27069	0.74305	0.74486	0	Uiso	1
C50	C	0.30751	0.81135	0.72192	0	Uiso	1
C51	C	0.27671	0.84906	0.72403	0	Uiso	1
C52	C	0.30324	0.70336	0.74347	0	Uiso	1
N53	N	0.26689	0.63673	0.74352	0	Uiso	1
C54	C	0.62493	0.90318	0.77308	0	Uiso	1
C55	C	-0.29975	0.93476	0.77308	0	Uiso	1
C56	C	0.59202	0.94570	0.77308	0	Uiso	1
C57	C	0.51656	0.91260	0.77308	0	Uiso	1
C58	C	0.48057	0.95223	0.77308	0	Uiso	1
C59	C	0.52315	1.02767	0.77308	0	Uiso	1
C60	C	0.59842	1.05877	0.77308	0	Uiso	1
C61	C	0.63105	1.01599	0.77308	0	Uiso	1
H62	H	0.03671	1.21264	0.66954	0	Uiso	1
H63	H	-0.03514	1.08649	0.66510	0	Uiso	1
H64	H	0.12538	1.06972	0.92510	0	Uiso	1
H65	H	0.19848	1.19529	0.92039	0	Uiso	1
H66	H	0.17135	0.65556	0.78868	0	Uiso	1
H67	H	0.36477	0.83717	0.70072	0	Uiso	1
H68	H	0.30787	0.90639	0.70372	0	Uiso	1
H69	H	-0.26627	0.99207	0.75615	0	Uiso	1
H70	H	0.48409	0.85477	0.77308	0	Uiso	1
H71	H	0.42262	0.92465	0.77308	0	Uiso	1
H72	H	0.49796	1.06030	0.77308	0	Uiso	1
H73	H	0.63227	1.11646	0.77308	0	Uiso	1
H74	H	-0.31115	1.04100	0.77308	0	Uiso	1

N75	N	0.33333	0.66667	0.29504	0	Uiso	1
N76	N	0.00000	1.00000	0.79504	0	Uiso	1

Supplementary Table 10. Atomic Coordinates for Simulated Structure of TFPA-TPB-COF-Q in AA Stacking Model.

C1	C	-0.73768	-0.37424	0.32109	0	Uiso	1
C2	C	-0.76834	-0.43825	0.17977	0	Uiso	1
C3	C	-0.83635	-0.47770	0.18213	0	Uiso	1
C4	C	-0.87530	-0.45359	0.32064	0	Uiso	1
C5	C	-0.84570	-0.38987	0.46007	0	Uiso	1
C6	C	-0.77765	-0.35092	0.46254	0	Uiso	1
C7	C	-0.94655	-0.49577	0.31768	0	Uiso	1
N8	N	-0.98493	-0.47363	0.40942	0	Uiso	1
C9	C	-1.05470	-0.51344	0.41361	0	Uiso	1
C10	C	-1.08601	-0.58138	0.36035	0	Uiso	1
C11	C	-1.15419	-0.61888	0.36615	0	Uiso	1
C12	C	-1.19223	-0.58923	0.42387	0	Uiso	1
C13	C	-1.16059	-0.52129	0.47729	0	Uiso	1
C14	C	-1.09238	-0.48378	0.47316	0	Uiso	1
C15	C	-1.26450	-0.62889	0.42656	0	Uiso	1
C16	C	-1.29599	-0.69738	0.42647	0	Uiso	1
C17	C	-0.57053	-0.95580	0.36035	0	Uiso	1
C18	C	-0.60816	-1.03112	0.36035	0	Uiso	1
C19	C	-0.61421	-0.92558	0.29138	0	Uiso	1
C20	C	-0.57895	-0.85031	0.29138	0	Uiso	1
C21	C	-0.61931	-0.81542	0.29138	0	Uiso	1
C22	C	-0.69462	-0.85176	0.29138	0	Uiso	1
C23	C	-0.72813	-0.92694	0.29138	0	Uiso	1
C24	C	-0.68694	-0.96096	0.29138	0	Uiso	1
H25	H	-0.73479	-0.45820	0.05723	0	Uiso	1
H26	H	-0.86209	-0.53237	0.06628	0	Uiso	1
H27	H	-0.87998	-0.37004	0.57548	0	Uiso	1
H28	H	-0.75180	-0.29686	0.58495	0	Uiso	1
H29	H	-1.18127	-0.67787	0.32098	0	Uiso	1
H30	H	-1.19283	-0.49477	0.52769	0	Uiso	1
H31	H	-1.06532	-0.42491	0.52153	0	Uiso	1
H32	H	-1.26372	-0.72484	0.42632	0	Uiso	1
H33	H	-0.66769	-1.06104	0.38748	0	Uiso	1
H34	H	-0.51919	-0.81856	0.29138	0	Uiso	1
H35	H	-0.59161	-0.75568	0.29138	0	Uiso	1
H36	H	-0.72346	-0.82083	0.29138	0	Uiso	1

H37	H	-0.78781	-0.96001	0.29138	0	Uiso	1
H38	H	-0.71395	-1.02066	0.29138	0	Uiso	1
N39	N	-0.66667	-0.33333	0.32106	0	Uiso	1

Supplementary Table 11. Atomic Coordinates for Simulated Structure of TFPA-TPB-COF-Q in AB Stacking Model.

C1	C	-0.07101	0.95910	0.16055	0	Uiso	1
C2	C	-0.10167	0.89508	0.08988	0	Uiso	1
C3	C	-0.16969	0.85563	0.09106	0	Uiso	1
C4	C	-0.20863	0.87974	0.16032	0	Uiso	1
C5	C	-0.17903	0.94347	0.23004	0	Uiso	1
C6	C	-0.11098	0.98242	0.23127	0	Uiso	1
C7	C	-0.27988	0.83757	0.15884	0	Uiso	1
N8	N	-0.31826	0.85970	0.20471	0	Uiso	1
C9	C	0.61197	0.81989	0.20680	0	Uiso	1
C10	C	0.58066	0.75195	0.18017	0	Uiso	1
C11	C	0.51248	0.71445	0.18308	0	Uiso	1
C12	C	0.47443	0.74410	0.21194	0	Uiso	1
C13	C	0.50608	0.81204	0.23864	0	Uiso	1
C14	C	0.57429	0.84955	0.23658	0	Uiso	1
C15	C	0.40217	0.70444	0.21328	0	Uiso	1
C16	C	0.37068	0.63596	0.21324	0	Uiso	1
C17	C	0.09613	0.37753	0.18017	0	Uiso	1
C18	C	0.05850	1.30221	0.18017	0	Uiso	1
C19	C	0.05245	0.40775	0.14569	0	Uiso	1
C20	C	0.08772	0.48302	0.14569	0	Uiso	1
C21	C	0.04736	0.51791	0.14569	0	Uiso	1
C22	C	-0.02795	0.48157	0.14569	0	Uiso	1
C23	C	-0.06146	0.40640	0.14569	0	Uiso	1
C24	C	-0.02027	0.37238	0.14569	0	Uiso	1
H25	H	-0.06813	0.87513	0.02862	0	Uiso	1
H26	H	-0.19543	0.80096	0.03314	0	Uiso	1
H27	H	-0.21331	0.96329	0.28774	0	Uiso	1
H28	H	-0.08514	1.03648	0.29248	0	Uiso	1
H29	H	0.48540	0.65547	0.16049	0	Uiso	1
H30	H	0.47383	0.83857	0.26384	0	Uiso	1
H31	H	0.60135	0.90843	0.26076	0	Uiso	1
H32	H	0.40295	0.60850	0.21316	0	Uiso	1
H33	H	-0.00102	1.27229	0.19374	0	Uiso	1
H34	H	0.14747	0.51478	0.14569	0	Uiso	1
H35	H	0.07505	0.57765	0.14569	0	Uiso	1
H36	H	-0.05680	0.51250	0.14569	0	Uiso	1

H37	H	-0.12114	0.37332	0.14569	0	Uiso	1
H38	H	-0.04728	1.31267	0.14569	0	Uiso	1
C39	C	0.26232	0.62576	0.66055	0	Uiso	1
C40	C	0.23166	0.56175	0.58988	0	Uiso	1
C41	C	0.16365	0.52230	0.59106	0	Uiso	1
C42	C	0.12470	0.54641	0.66032	0	Uiso	1
C43	C	0.15430	0.61013	0.73004	0	Uiso	1
C44	C	0.22235	0.64908	0.73127	0	Uiso	1
C45	C	0.05345	0.50423	0.65884	0	Uiso	1
N46	N	0.01507	0.52637	0.70471	0	Uiso	1
C47	C	0.94530	0.48656	0.70680	0	Uiso	1
C48	C	0.91399	0.41862	0.68017	0	Uiso	1
C49	C	0.84581	0.38112	0.68308	0	Uiso	1
C50	C	0.80777	0.41077	0.71194	0	Uiso	1
C51	C	0.83941	0.47871	0.73864	0	Uiso	1
C52	C	0.90762	0.51622	0.73658	0	Uiso	1
C53	C	0.73550	0.37111	0.71328	0	Uiso	1
C54	C	0.70401	0.30262	0.71324	0	Uiso	1
C55	C	0.42947	0.04420	0.68017	0	Uiso	1
C56	C	0.39184	0.96888	0.68017	0	Uiso	1
C57	C	0.38579	0.07442	0.64569	0	Uiso	1
C58	C	0.42105	0.14969	0.64569	0	Uiso	1
C59	C	0.38069	0.18458	0.64569	0	Uiso	1
C60	C	0.30538	0.14824	0.64569	0	Uiso	1
C61	C	0.27187	0.07306	0.64569	0	Uiso	1
C62	C	0.31306	0.03904	0.64569	0	Uiso	1
H63	H	0.26521	0.54180	0.52862	0	Uiso	1
H64	H	0.13791	0.46763	0.53314	0	Uiso	1
H65	H	0.12002	0.62996	0.78774	0	Uiso	1
H66	H	0.24820	0.70314	0.79248	0	Uiso	1
H67	H	0.81873	0.32213	0.66049	0	Uiso	1
H68	H	0.80717	0.50523	0.76384	0	Uiso	1
H69	H	0.93468	0.57509	0.76076	0	Uiso	1
H70	H	0.73628	0.27516	0.71316	0	Uiso	1
H71	H	0.33231	0.93896	0.69374	0	Uiso	1
H72	H	0.48081	0.18144	0.64569	0	Uiso	1
H73	H	0.40839	0.24432	0.64569	0	Uiso	1
H74	H	0.27654	0.17917	0.64569	0	Uiso	1

H75	H	0.21219	0.03999	0.64569	0	Uiso	1
H76	H	0.28605	0.97934	0.64569	0	Uiso	1
N77	N	0.00000	1.00000	0.16053	0	Uiso	1
N78	N	0.33333	0.66667	0.66053	0	Uiso	1

Supplementary References

1 Liu, J., Cao, C., Liu, X., Zheng, L., Yu, X., Zhang, Q., Gu, L., Qi, R. & Song, W. Direct observation of metal oxide nanoparticles being transformed into metal single atoms with oxygen-coordinated structure and high-loadings. *Angew. Chem. Int. Ed.* **60**, 15248-15253 (2021).

2 Li, X., Zhang, C., Cai, S., Lei, X., Altoe, E., Hong, F., Urban, J. J., Ciston, J., Chan, E. M. & Liu, Y. Facile transformation of imine covalent organic frameworks into ultrastable crystalline porous aromatic frameworks. *Nat. Chem.* **9**, 2998 (2018).

3 Li, X.-T., Zou, J., Wang, T.-H., Ma, H.-C., Chen, G.-J. & Dong, Y.-B. Construction of covalent organic frameworks via three-component one-pot Strecker and Povarov reactions. *J. Am. Chem. Soc.* **142**, 6521-6526 (2020).

4 Zhao, Y., Sui, Z., Chang, Z., Wang, S., Liang, Y., Liu, X., Feng, L., Chen, Q. & Wang, N. A trifluoromethyl-grafted ultra-stable fluorescent covalent organic framework for adsorption and detection of pesticides. *J. Mater. Chem. A* **8**, 25156-25164 (2020).

5 Zhao, Y., Yang, Y., Xia, T., Tian, H., Li, Y., Sui, Z., Yuan, N., Tian, X. & Chen, Q. Pyrimidine-functionalized covalent organic framework and its cobalt complex as an efficient electrocatalyst for oxygen evolution reaction. *ChemSusChem* **14**, 4556-4562 (2021).

6 Ding, L.-G., Yao, B.-J., Wu, W.-X., Yu, Z.-G., Wang, X.-Y., Kan, J.-L. & Dong, Y.-B. Metalloporphyrin and ionic liquid-functionalized covalent organic frameworks for catalytic CO_2 cycloaddition via visible-light-induced photothermal conversion. *Inorg. Chem.* **60**, 12591-12601 (2021).

7 Yang, Y., Yu, L., Chu, T., Niu, H., Wang, J., & Cai, Y. Constructing chemical stable 4-carboxyl-quinoline linked covalent organic frameworks via Doebner reaction for nanofiltration. *Nat. Commun.* **13**, 2615 (2022).

8 Li, X.-T., Zou, J., Yu, Q., Liu, Y., Li, J.-R., Li, M.-J., Ma, H.-C., Chen, G.-J. & Dong, Y.-B. Construction of acid-base bifunctional covalent organic frameworks via Doebner reaction for catalysing cascade reaction. *Chem. Commun.* **58**, 2508-2511 (2022).

9 Ren, X.-R., Bai, B., Zhang, Q., Guo, Y., Wan, L.-J. & Wang, D. Constructing stable chromenoquinoline-based covalent organic frameworks via intramolecular Povarov reaction. *J. Am. Chem. Soc.* **144**, 2488-2494 (2022).

10 Zhao, X., Pang, H., Huang, D., Liu, G., Hu, J. & Xiang, Y. Construction of ultrastable nonsubstituted quinoline-bridged covalent organic frameworks via rhodium-catalyzed dehydrogenative annulation. *Angew. Chem. Int. Ed.* **61**, e202208833 (2022).

11 Chen, R., Zhao, J., Yu, Z., Cong, M., Wang, Y., Wang, M., Li, G., Li, Z. & Zhao, Y. Post-synthetic fully π-conjugated three-dimensional covalent organic frameworks for high-performance lithium storage. *ACS Appl. Mater. Interfaces* **15**, 830-837 (2022).

12 Das, P., Chakraborty, G., Roeser, J., Vogl, S., Rabeah, J. & Thomas, A. Integrating bifunctionality and chemical stability in covalent organic frameworks via one-pot multicomponent reactions for solar-driven H_2O_2 production. *J. Am. Chem. Soc.* **145**, 2975-2984 (2023).

13 Xiao, Z., Nie, X., Li, Y., Nie, Y., Lu, L. & Tian, X. Boric acid functional fluorescent covalent–organic framework for sensitive and selective visualization of CH_3Hg^+. *ACS Appl. Mater. Interfaces* **15**, 9524-9532 (2023).

14 Das, P., Roeser, J. & Thomas, A. Solar light driven H_2O_2 production and selective oxidations using a covalent organic framework photocatalyst prepared by a multicomponent reaction. *Angew. Chem. Int. Ed.* **62**, e202304349 (2023).

15 Pang, H., Huang, D., Zhu, Y., Zhao, X. & Xiang, Y. One-pot cascade construction of nonsubstituted quinoline-bridged covalent organic frameworks. *Chem. Sci.* **14**, 1543-1550 (2023).

16 Kou, M., Wang, Y., Xu, Y., Ye, L., Huang, Y., Jia, B., Li, H., Ren, J., Deng, Y., Chen, J., Zhou, Y., Lei, K., Wang, L., Liu, W., Huang, H. & Ma, T. Molecularly engineered covalent organic frameworks for hydrogen peroxide photosynthesis. *Angew. Chem. Int. Ed.* **61**, e202200413 (2022).

17 Mo, Y., Wu, X., Qin, C., Chen, J., Zhao, Y., Jiang, L., Zhang, C., Yuan, X., Ang, E. H. & Wang, H. Linkage microenvironment of azoles-related covalent organic frameworks precisely regulates photocatalytic generation of hydrogen peroxide. *Angew. Chem. Int. Ed.* **62**, e202309480 (2023).

18 Tan, F., Zheng, Y., Zhou, Z., Wang, H., Dong, X., Yang, J., Ou, Z., Qi, H., Liu, W., Zheng, Z. & Chen, X. Aqueous synthesis of covalent organic frameworks as photocatalysts for hydrogen peroxide production. *CCS Chem.* **4**, 3751-3761 (2022).

19 Yang, J., Acharjya, A., Ye, M.-Y., Rabeah, J., Li, S., Kochovski, Z., Youk, S., Roeser, J., Grüneberg, J., Penschke, C., Schwarze, M., Wang, T., Lu, Y., Krol, R. V. D., Oschatz, M., Schomäcker, R., Saalfrank, P. & Thomas, A. Protonated imine-linked covalent organic frameworks for photocatalytic hydrogen evolution. *Angew. Chem. Int. Ed.* **60**, 19797-19803 (2021).

20 Reichle, A., Sterzel, H., Kreitmeier, P., Fayad, R., Castellano, F. N., Rehbein, J. & Reiser, O. Copper(II)-photocatalyzed decarboxylative oxygenation of carboxylic acids. *Chem. Commun.* **58**, 4456-4459 (2022).
